# Luminescent Ln(III)-Metallopeptide Sensors for Monitoring *Pseudomonas aeruginosa* Elastase B Activity in Complex Biological Media

**DOI:** 10.1021/acssensors.4c00986

**Published:** 2024-09-06

**Authors:** Rosalía Sánchez-Fernández, Martín Sandá-Ares, Nerea Lamas, Trinidad Cuesta, José Luis Martínez, Paco Fernandez-Trillo, Elena Pazos

**Affiliations:** † CICA−Centro Interdisciplinar de Química e Bioloxía and Departamento de Química, Facultade de Ciencias, Universidade da Coruña. Campus de Elviña, 15071 A Coruña, Spain; ‡ Centro Nacional de Biotecnología, CSIC, Darwin 3, 28049 Madrid, Spain

**Keywords:** lanthanides, metallopeptides, luminescent sensors, protease activity, LasB, *P. aeruginosa*

## Abstract

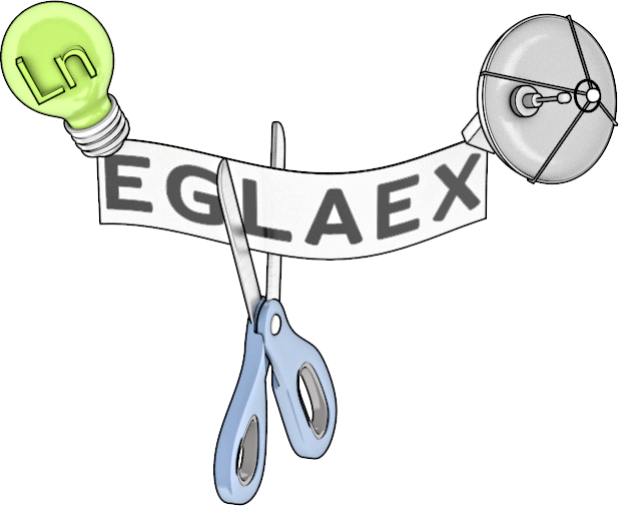

The detection and monitoring of *Pseudomonas aeruginosa* and its virulence factors, such as the LasB protease, are crucial for managing bacterial infections. Traditional fluorescent sensors for this protease face limitations in bacterial cultures due to interference from pigments like pyoverdine secreted by this opportunistic pathogen. We report here a Ln(III)-metallopeptide that combines a DO3A-Ln(III) complex and a sensitizing unit via a short peptide sequence as a simple, tunable, and selective probe for detecting *P. aeruginosa*’s LasB. The probe’s luminescence switches off in the presence of *P. aeruginosa*’s secretome due to LasB cleavage but remains stable in other bacterial environments, such as non-LasB-secreting *P. aeruginosa* strains or *E. coli* cultures. It also resists degradation by other proteases, like human leukocyte elastase and trypsin, and remains stable in the presence of bioanalytes related to *P. aeruginosa* infections, such as glutathione, H_2_O_2_, and pyocyanin, and in complex media like FBS. Importantly, time-gated experiments completely remove the background fluorescence of *P. aeruginosa* pigments, thus demonstrating the potential of the developed Ln(III)-metallopeptide for real-time monitoring of LasB activity in bacterial cultures.

*Pseudomonas aeruginosa* is a frequent cause of infection, especially in hospital-acquired infections or in immunocompromised patients, such as those with chronic obstructive pulmonary disease or cystic fibrosis.^1,2^ Given its low antibiotic susceptibility, it has been included by the World Health Organization in the global priority list of pathogens.^3,4^ However, its pathogenicity is not only due to its antibiotic resistance but also its extensive arsenal of extracellular and cell-associated virulence factors that allow it to adapt to different environmental conditions. Among these virulence factors, *P. aeruginosa* secretes various proteases critical for invasion in acute infections, with elastase B (LasB) being the most abundant protease and the main extracellular virulence factor.^5,6^ Therefore, fast and simple detection of virulent strains of *P. aeruginosa* by identifying these virulence factors is of great interest to manage bacterial contamination and initiate treatment.

Several assays for *P. aeruginosa* proteases, including LasB, have been developed.^7^ Nevertheless, in most cases, they do not allow real-time monitoring of enzymatic activity. In this context, luminescent techniques are very attractive because they are sensitive, simple, and nondestructive. Not surprisingly, fluorescent probes based on organic fluorophores have been reported to detect *P. aeruginosa* proteases.^8−12^ These probes are highly sensitive in detecting protease activity with limits of detection (LODs) in the low nanomolar range.^11,12^ However, their emission falls within the blue-green region, similar to the fluorescent siderophores pyoverdine and pyochelin secreted by *P. aeruginosa*,^13,14^ and other fluorescent compounds inherent to biological samples, as reported by Schönherr.^12^ This similarity limits the effectiveness of the reported organic probes to monitor protease activity in *P. aeruginosa* cultures.

In contrast to organic fluorophores, lanthanide ions have unique photophysical properties, including narrow emission bands in the visible and near-infrared. Most importantly, Tb(III) and Eu(III) ions exhibit long lifetimes on the order of milliseconds, which allows the removal of the fluorescence background signal from biological samples using time-resolved luminescence.^15^ Although many lanthanide complexes have been described for monitoring enzyme activity,^16^ there are only a limited number of lanthanide-based probes for proteases, including detection of leucine aminopeptidase,^17,18^ calpain I,^18^ and caspases 1, 3, and 6.^19−21^

We report here a new and simple sensing strategy to monitor LasB activity, and thus detect virulent strains of *P. aeruginosa*, using luminescent Ln(III)-metallopeptides. The sensing mechanism is based on the energy transfer from the sensitizing unit (antenna) to the DO3A-Ln(III) complex, which are joined by a LasB substrate (Figure 1). In this way, the presence of LasB leads to the cleavage of the peptide sequence, with subsequent loss of emission. The flexibility of this molecular design lets us easily change the Ln(III) ion and the antenna to give both green and red-emitting probes. Moreover, time-gated luminescence with *P. aeruginosa* supernatants allows us to monitor the emission of these probes in real time in the presence of the fluorophores secreted by the bacteria. Importantly, the reported probes are selective for LasB and related proteins and are not degraded by mutant strains of *P. aeruginosa* and other microorganisms that do not secrete LasB.

**Figure 1:**
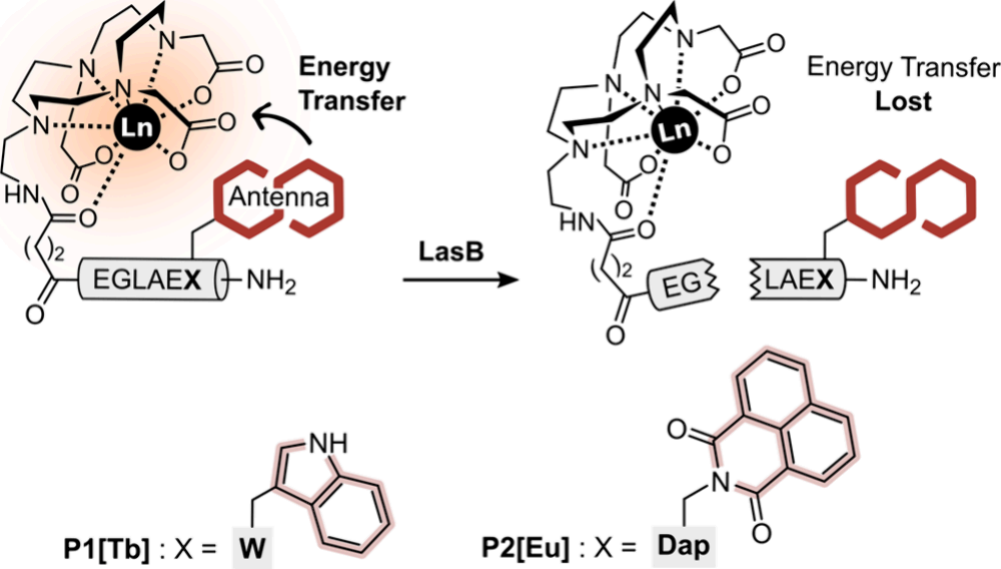
Schematic representation of the sensing strategy used to monitor LasB activity.

The design of the flexible Ln(III)-based probes for LasB (Figure 1) started with the peptide sequence GLA. This peptide sequence is selectively degraded by LasB and related proteins,^22^ and has been previously used to develop LasB-responsive nanoparticles.^23,24^ Glutamic acids were introduced on each side of this sequence to increase the solubility of the metallopeptides in water. More importantly, we added a DO3A-Tb(III) complex at the *N*-terminus and a tryptophan (Trp) residue as a sensitizing unit at the *C*-terminus of the peptide sequence,^25−28^ to give the green-emitting metallopeptide **P1[Tb]**. This way, the peptide sequence EGLAEW was synthesized following standard Fmoc/*t*Bu solid phase synthesis protocols (Scheme S1 in the Supporting Information). Next, the free *N*-terminus of the peptide sequence was reacted with succinic anhydride to then attach a DO3A-ethylamino derivative through an amide bond. The resulting peptide **P1** was fully deprotected and cleaved from the solid support with TFA and then purified by reversed-phase HPLC. Finally, **P1[Tb]** was prepared by incubating a **P1** solution in HEPES buffer (10 mM HEPES, pH 8) with TbCl_3_. ESI-MS revealed the presence of the targeted metallopeptide (*m*/*z* = 665.7411 [M+2H]^2+^), demonstrating the success of the functionalization (Figure S2). The time-gated luminescence spectrum of **P1[Tb]** showed the characteristic Tb(III) emission bands centered at 489, 544, 585, and 620 nm upon excitation of the Trp residue at 282 nm (Figure S5), verifying the formation of the desired metallopeptide complex.

With the targeted peptide **P1[Tb]** in hand, the next step was to demonstrate that the presence of the DO3A-Tb(III) complex and the terminal Trp residue did not inhibit cleavage of the GLA sequence. Since LasB is a Ca(II)-dependent enzyme, we first incubated a 10 μM **P1[Tb]** solution in HEPES buffer with 1 mM CaCl_2_ to confirm that the Ca(II) ion did not compete with Tb(III) complexation (Figure S6). Then, a 10 μM **P1[Tb]** and 1 mM CaCl_2_ solution in HEPES buffer was incubated with 60.6 nM LasB (∼2 μg/mL), and the emission intensity at 544 nm was monitored over time. This emission intensity decreased with time and was almost completely turned off after 3 h (Figure 2A and Figure S6), suggesting that **P1[Tb]** was degraded by LasB, thereby stopping the energy transfer from the indole antenna to the DO3A-Tb(III) complex (Figure 1).^22^ To examine the selectivity of our probe, we incubated **P1[Tb]** (10 μM) with other proteases, in particular human leukocyte elastase (HLE, ∼1 μg/mL), part of our immune response to infection and inflammation, and trypsin (≈ 3.5 μg/mL), commonly used in cell biology and proteomics. Both proteases could interfere with our probe in assays involving human samples and/or cell cultures. As shown in Figures S7 and S8, no changes in the luminescence intensity of **P1[Tb]** were observed after 20 h in the presence of either of these enzymes, confirming that the probe was not degraded by any of these proteases.

**Figure 2:**
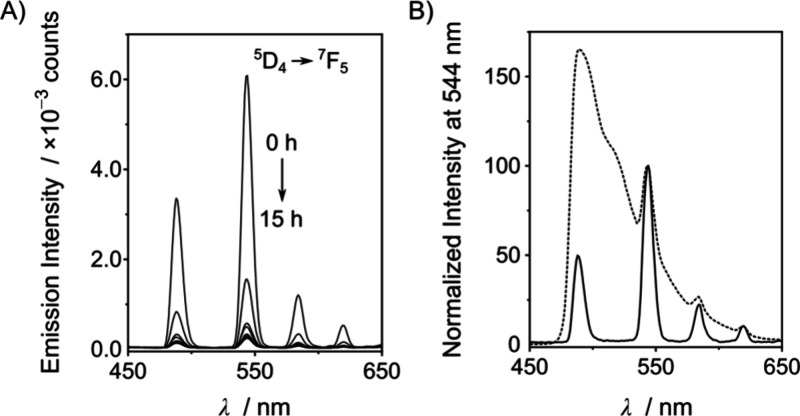
(A) Luminescence spectra of 10 μM **P1[Tb]**, 1 mM CaCl_2_ and 60.6 nM LasB in 10 mM HEPES, pH 8, at 37 °C between 0 and 15 h after the addition of the enzyme. (B) Steady-state (- - -) and time-gated (—) emission spectra at 37 °C of 10 μM **P1[Tb]** and 1 mM CaCl_2_ in 10 mM HEPES, pH 8, in the presence of the supernatant from the LasB2 mutant strain of *P. aeruginosa*. The spectra have been normalized to the intensity at 544 nm, corresponding to the Tb(III) transition ^5^D_4_ → ^7^F_5_.

With our probe working as intended, the next step was to determine the kinetics of its degradation in the presence of LasB. To this end, **P1[Tb]** solutions at different concentrations were incubated in the presence of LasB, to monitor the rate of reaction under these conditions (Figure S9). Unfortunately, the Michaelis–Menten constant (*K*_m_) could not be determined due to the low **P1[Tb]** concentration when compared to the estimated *K*_m_. However, based on the simple “hit-and-run” mechanism shown in Scheme S3,^29^ we could calculate an apparent rate constant *k*_sub_ = (0.438 ± 0.007) μM^–1^min^–1^, which is equivalent to the specificity constant *k*_cat_/*K*_m_. The calculated value for **P1[Tb]** was lower than that of previously reported LasB probes.^8,9,12^ We also calculated the limit of quantification (LOQ) and the LOD for LasB after 1 h of incubation (19.3 nM and 6.7 nM, respectively, Figures S10 and S11).^30^ Interestingly, the LOD for our probe **P1[Tb]** was approximately 2.5-fold better than that reported using a hydrogel sensor.^12^

As mentioned, our goal with this work was to develop a probe that could be used for real-time monitoring of bacterial cultures, thus overcoming the limitation of previous FRET-based probes. To this end, we recorded the luminescence spectra of **P1[Tb]** when aliquots of supernatants from *P. aeruginosa* cultures were present. Specifically, we used supernatants from the wild-type strain PA14 and two isogenic mutants that could not produce LasB.^31^ Elastase activity for these strains was measured using elastin-congo red as a substrate and, as expected, confirmed that the wild-type parental strain produced LasB. In contrast, the two *ΔlasB* mutants (LasB1 and LasB2) did not produce this protease (Table S2).^32^ Using the supernatant from one of these *ΔlasB* mutants, we could demonstrate that time-gated luminescence eliminated the fluorescent background signal characteristic of the *P. aeruginosa* cultures (Figure 2B). This way, we could clearly see the characteristic signals at 489, 544, 585, and 620 nm of the Tb(III) metallopeptide probe **P1[Tb]** (Figure 2B, solid line), which is not the case in the steady-state spectrum (Figure 2B, dashed line), highlighting the advantages of lanthanide complexes as emitting units.

Having demonstrated that time-gated luminescence removed the background luminescence of *P. aeruginosa* cultures, the next step was to use **P1[Tb]** to monitor the LasB activity of *P. aeruginosa* cultures. To this end, a 10 μM **P1[Tb]** and 1 mM CaCl_2_ solution in HEPES buffer was incubated at 37 °C with 10 μL of supernatant from a *P. aeruginosa* PA14 culture, the wild-type strain that secretes LasB. Similar to the pure enzyme, the luminescence intensity decreased over time and was practically switched off after 4 h (Figure 3 and Figure S12). More importantly, when we incubated **P1[Tb]** in the presence of 10 μL of the supernatant from cultures of the two mutant *P. aeruginosa* strains that do not secrete LasB (Figure 3B) or with an *E. coli* supernatant (Figure S14), no changes in the luminescence of **P1[Tb]** were observed over time. This lack of activity demonstrated that LasB-producing microorganisms selectively degraded this probe. In addition, HPLC-MS of **P1[Tb]** in the absence and presence of *P. aeruginosa* cultures showed that **P1[Tb]** was cleaved at the G-L bond, as previously reported for LasB and related enzymes.^22^ In contrast, it remained intact in the presence of the supernatants from the LasB-deficient *P. aeruginosa* mutants (Figure S13). To unequivocally validate the cleavage of **P1[Tb]** by LasB in the presence of the wild-type *P. aeruginosa* supernatant, we repeated this experiment while introducing EDTA, an inhibitor of LasB.^33^ In this case, the luminescence of **P1[Tb]** is reduced by approximately 30% 3 h after its addition. In contrast, in the absence of EDTA, the observed luminescence was reduced by approximately 90%, thus corroborating its cleavage by LasB (Figure S15).

**Figure 3:**
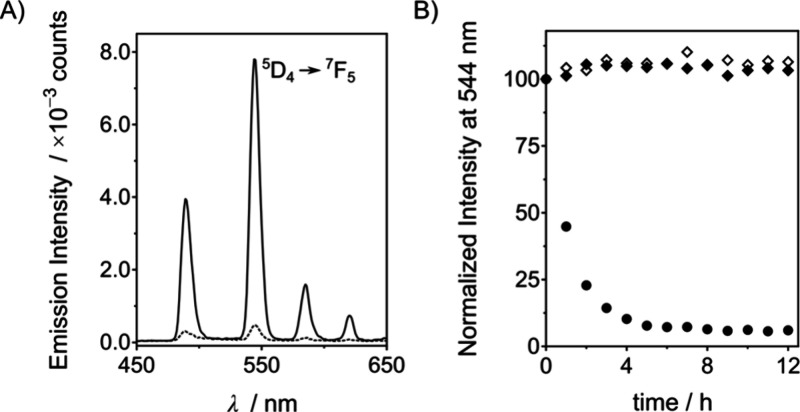
(A) Luminescence spectra at 37 °C of 10 μM **P1[Tb]** and 1 mM CaCl_2_ in 10 mM HEPES, pH 8, at 0 h (—) and 12 h after the addition of 10 μL of supernatant from the culture of a *P. aeruginosa* strain secreting LasB (- - -). (B) Luminescence intensity at 544 nm over time of 10 μM **P1[Tb]** and 1 mM CaCl_2_ in 10 mM HEPES, pH 8, at 37 °C in the presence of 10 μL of *P. aeruginosa* supernatants from wild-type (●), LasB1 mutant (◆), and LasB2 mutant (◇) strains.

To confirm the suitability of **P1[Tb]** to monitor LasB activity in bacterial cultures, we investigated its stability in the presence of relevant concentrations of other bioanalytes. We specifically explored the effects of glutathione, a thiol-containing tripeptide crucial for oxidative stress management by *P. aeruginosa* during infection,^34^ H_2_O_2_, a reactive oxygen species (ROS) produced by the host immune system to combat *P. aeruginosa* infection,^35,36^ and pyocyanin, a virulence factor in *P. aeruginosa* that is involved in the generation of ROS during infection.^37^ As anticipated, incubation with pyocyanin reduced the intensity of the bands associated with **P1[Tb]** by approximately 20%, as a result of the strong absorption of this virulence factor in the UV region. Despite this reduced emission, the probe remained active, and we could see a gradual decrease in the luminescence following the addition of the *P. aeruginosa* supernatant (Figure S18). Conversely, glutathione and H_2_O_2_ did not affect the luminescence of **P1[Tb],** which remained constant for 20 h after the addition of both molecules (Figures S16 and S17, respectively). Finally, **P1[Tb]** was incubated in 1.2% fetal bovine serum (FBS) without compromising its luminescence for at least 12 h. The subsequent addition of the *P. aeruginosa* supernatant led to a gradual reduction in the luminescence emission of **P1[Tb]**, showcasing the probe’s remarkable ability to monitor LasB activity effectively in complex biological media (Figure S19).

To demonstrate the versatility of the molecular design of **P1[Tb]**, we replaced the Tb(III) ion with Eu(III) and the indole antenna with a naphthalimide moiety, known to sensitize Eu(III) ions (Figure 1).^38^ This modification resulted in the metallopeptide **P2[Eu],** which emitted in the red region of the visible spectrum, avoiding overlap with the background fluorescence emission of *P. aeruginosa* cultures. Consequently, this red-emitting probe should be more practical for use in biomedical laboratories that may not have time-resolved experimental capabilities. **P2[Eu]** was synthesized analogously to **P1[Tb]**, with the Trp residue replaced with an Alloc-protected 2,3-diaminopropanoic acid residue (Dap(Alloc)), to which 1,8-naphthalic anhydride was coupled on the solid support to the orthogonally deprotected Dap side chain (Scheme S2). The formation of the Eu(III) metallopeptide **P2[Eu]** was confirmed by ESI-MS (Figure S4) and luminescence, with the characteristic Eu(III) emission bands at 578, 590, 615, 651, and 697 nm upon excitation at 344 nm (Figure S5).

Next, we studied **P2[Eu]** cleavage kinetics with LasB. When this probe was incubated with LasB we observed similar response-time profiles to those obtained with **P1[Tb]** (Figure S20), and we calculated an apparent rate constant *k*_sub_ = (0.239 ± 0.005) μM^–1^min^–1^. The decrease in *k*_sub_ indicates that substituting Trp with the naphthalimide moiety affects the interaction of **P2[Eu]** with LasB. This observation was supported by the calculated LOQ = 28.5 nM and LOD = 14.9 nM after 1 h of incubation (Figures S21 and S22), which were higher than those obtained for **P1[Tb]**. Importantly, the obtained LOD was still better than that previously reported.^12^

We then incubated **P2[Eu]** with the *P. aeruginosa* supernatants. As expected, the luminescence intensity at 615 nm of **P2[Eu]** in the presence of the wild-type *P. aeruginosa* supernatant decreased with time and was almost switched off after 6 h (Figure 4 and S23). In contrast, the luminescence remained largely unchanged in the presence of the supernatants from the two LasB-deficient *P. aeruginosa* strains (Figure 4B). Additionally, the presence of **P2[Eu]** after treatment with the supernatants from the *ΔlasB* strains, as well as its cleavage by LasB in the *P. aeruginosa* PA14 supernatant, was confirmed by HPLC-MS (Figure S24) as previously demonstrated for **P1[Tb]**. Similarly, we validated the selectivity of the **P2[Eu]** probe using HLE, trypsin, and an *E. coli* supernatant, finding again that the luminescence of **P2[Eu]** remained stable even after 20 h of incubation (Figures S25–S27).

**Figure 4:**
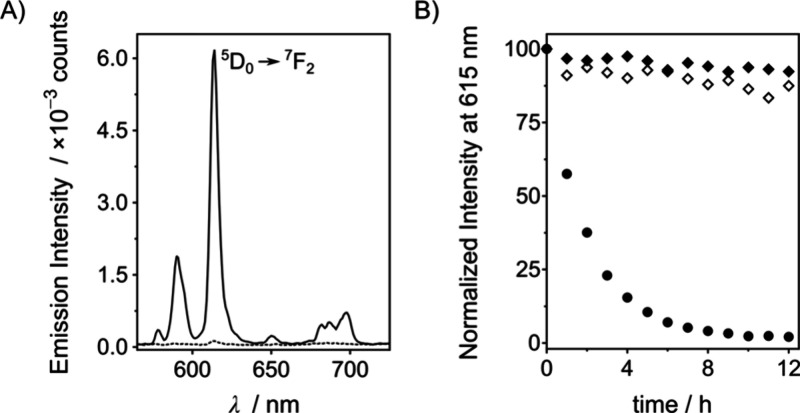
(A) Luminescent spectra at 37 °C of a 10 μM **P2[Eu]** and 1 mM CaCl_2_ solution in 10 mM HEPES, pH 8, at 0 h (—) and 12 h after the addition of 10 μL of a wild-type *P. aeruginosa* supernatant (- - -). (B) Luminescence intensity at 615 nm over time of 10 μM **P2[Eu]** and 1 mM CaCl_2_ in 10 mM HEPES, pH 8, at 37 °C in the presence of 10 μL of *P. aeruginosa* supernatants from cultures of wild-type (●), LasB1 mutant (◆), and LasB2 mutant (◇) strains.

Consistent with **P1[Tb]**, **P2[Eu]** remained active in the presence of pyocyanin, despite the effect this virulence factor has on the initial intensity of the luminescence emission (Figure S30). This red-emitting probe also showed remarkable stability in the presence of glutathione and H_2_O_2_, with no changes in the luminescence observed after 20 h of incubation with both molecules (Figures S28 and S29, respectively). Furthermore, the luminescence of **P2[Eu]** remained stable for 12 h, even in the presence of 10% FBS. The addition of the *P. aeruginosa* supernatant led again to a clear decrease in the luminescence of **P2[Eu]** over time, clearly demonstrating this probe’s capability for effective real-time monitoring of LasB activity in complex biological media (Figure S31). Crucially, when we compared the time-gated and steady-state spectra of **P2[Eu]** in the presence of the supernatant from the *P. aeruginosa* LasB2 mutant (Figure S32), we confirmed that the characteristic narrow emission band at 615 nm from Eu(III) ions barely overlaps with the fluorescent background signal from *P. aeruginosa* cultures. As anticipated, this minimal overlap confirms that **P2[Eu]** metallopeptide is highly effective for monitoring LasB activity, and should be of great value in settings where instruments cannot perform time-resolved luminescence experiments.

In conclusion, we present here the first lanthanide-based probes to monitor the expression of virulence factors in *P. aeruginosa*, specifically its main extracellular virulence factor LasB. We employed a modular molecular design to prepare both green-emitting **P1[Tb]** and red-emitting **P2[Eu]** probes. Both probes exhibited selective luminescence quenching in the presence of LasB but not in the presence of *P. aeruginosa* strains or microorganisms that do not secrete LasB. We further demonstrate the specificity of these probes, showing no degradation by other proteases (i.e., human leukocyte elastase and trypsin). The probes remained stable in the presence of bioanalytes associated with *P. aeruginosa* infection (i.e., glutathione, pyocyanin or H_2_O_2_) and also in complex media (i.e., FBS). Under these conditions, the probes remained active and achieved LasB-mediated quenching, which was comparable to the quenching achieved in controlled environments. The probes developed in this work provide a significant advantage over commercial substrates used to assess *P. aeruginosa* elastase activity, such as the elastin-congo red, which requires long incubation times (up to 12–16 h according to the manufacturer’s protocol) and filtration/centrifugation steps,^7,39,40^ thus hampering real-time monitoring of the enzyme. Crucially, we have demonstrated that the fluorescent signal of the *P. aeruginosa* pigments was completely removed by using time-gated experiments. We strongly believe that the developed LasB probes should underpin the real-time monitoring of virulent strains of *P. aeruginosa* in bacterial cultures.

## Supporting Information Available

Materials and instrumentation, peptide synthesis, experimental procedures, analytical data, and luminescence assays (PDF)
