# Supplementary material for: Luminescent Ln(III)-Metallopeptide Sensors for Monitoring Pseudomonas aeruginosa Elastase B Activity in Complex Biological Media
Source: ACS Sens. 2024 Sep 6;9(10):5052–7. doi: 10.1021/acssensors.4c00986 (PMC11519908; doi:10.1021/acssensors.4c00986)
Supplement: Supplementary file 1 — se4c00986_si_001.pdf [file se4c00986_si_001.pdf]

Supporting Information

Luminescent Ln(III)-metallopeptide sensors for monitoring *Pseudomonas aeruginosa* Elastase B activity in complex biological media

Rosalía Sánchez-Fernández,[a] Martín Sandá-Ares,[a] Nerea Lamas,[a] Trinidad Cuesta,[b] José Luis Martínez,[b] Paco Fernandez-Trillo,[a]* and Elena Pazos [a]*

[a] CICA – Centro Interdisciplinar de Química e Bioloxía and Departamento de Química, Facultade de Ciencias. Universidade da Coruña. Campus de Elviña, 15071 A Coruña, Spain. [b] Centro Nacional de Biotecnología, CSIC, Darwin 3, 28049, Madrid, Spain.

E-mail: f.ftrillo@udc.es, elena.pazos@udc.gal

Contents

Materials 3

Instrumentation 3

Bacterial strains and culture conditions 4

Peptide synthesis 5

P1[Tb] metallopeptide 5

P2[Eu] metallopeptide 7

Luminescent spectra of **P1[Tb]** and **P2[Eu]** 9

Time course experiments of **P1[Tb]** with LasB 9

Determination of **P1[Tb]** specificity constant (k_{sub}) for LasB 11

Determination of the LOD and LOQ with **P1[Tb]** 11

Steady-state vs time-gated luminescence spectra of **P1[Tb]** in the presence of the supernatant from a LasB-deficient *P. aeruginosa* strain culture 12

Time course experiments of **P1[Tb]** metallopeptide with *P. aeruginosa* supernatants 13

Luminescence spectra of **P1[Tb]** in the presence of an *E. coli* supernatant 14

Luminescence spectra of **P1[Tb]** in the presence of the *P. aeruginosa* supernatant secreting LasB and EDTA 14

Luminescence spectra of **P1[Tb]** in the presence of glutathione (GSH) and H_2O_2 15

Luminescence spectra of **P1[Tb]** in the presence of pyocyanin and *P. aeruginosa* supernatant 16

Luminescence spectra of **P1[Tb]** in the presence of 1.2% FBS and *P. aeruginosa* supernatant 16

Determination of **P2[Eu]** specificity constant (k_{sub}) for LasB 17

Determination of the LOD and LOQ with **P2[Eu]** 18

Time course experiments of **P2[Eu]** metallopeptide with *P. aeruginosa* supernatants.............................. 19

Luminescence spectra of **P2[Eu]** in the presence of HLE, trypsin, and an *E. coli* supernatant.................. 20

Luminescence spectra of **P2[Eu]** in the presence of GSH and H_2O_2... 21

Luminescence spectra of **P2[Eu]** in the presence of pyocyanin and *P. aeruginosa* supernatant 22

Luminescence spectra of **P2[Eu]** in the presence of 10% FBS and *P. aeruginosa* supernatant 22

Steady-state vs time-gated luminescence spectra of **P2[Eu]** in the presence of the supernatant from a LasB-deficient *P. aeruginosa* strain culture 23

References 23

Materials

Amino acid derivatives and coupling agents were purchased from *Iris Biotech GmbH;* amino acids were purchased as protected Fmoc amino acids with the standard side chain protecting scheme: Fmoc-Gly-OH, Fmoc-Leu-OH, Fmoc-Ala-OH, Fmoc-Glu(O*t*Bu)-OH, and Fmoc-Trp(Boc)-OH, except for the orthogonally protected Fmoc-Dap(Alloc)-OH which was purchased from *BLDpharm*. *C*-terminal amide peptides were synthesized on a 0.1 mmol scale using 0.41 mmol/g loading H-Rink amide ChemMatrix resin from *Merck*. 1,8-Naphthalic anhydride was purchased from *TCI Europe.* LasB from *P. aeruginosa* was purchased from *Elastin Products Company* (Ref.#: PE961). Human leukocyte elastase was purchased from *Merck* (Ref.#: 324681). Trypsin was purchased as Trypsin Gold, Mass Spectrometry Grade from *Promega* (Ref.#: V5280). All other chemicals were purchased from *Fisher Scientific* and *Merck*. All solvents were synthesis grade, except for dimethylformamide (DMF), diisopropylethylamine (DIEA), and trifluoroacetic acid (TFA), which were peptide synthesis grade. Water was purified using a Milli-Q system (*Millipore*).

Instrumentation

Reversed phase HPLC analyses were performed using a Liquid Chromatograph system *Agilent* 1200 series. Reversed phase HPLC and Electrospray Ionization Mass Spectrometry (ESI/MS) analyses were performed using a Liquid Chromatograph Mass Spectrometer system, *Bruker* Elute UHPLC, connected to a mass spectrometer *Bruker* amaZon speed Toxtyper or an *Agilent* 1290 Infinity II LC system coupled to an *Agilent* 6546 Q-TOF mass spectrometer. In both cases, an Aeris analytical column (peptide XB-C18 stationary phase, 3.6 μm, 100 Å pore size, 150 × 2.1 mm) or a Luna analytical column (Omega Polar C18 stationary phase, 3 μm, 100 Å pore size, 150 × 2.1 mm), both from *Phenomenex*, were used. The standard method used for analytical HPLC was 5 → 95% MeCN, 0.04% TFA / H_2O, 0.04% TFA over 23 min. HPLC-MS quality solvents were used to prepare the eluents.

Concentrations were determined using a UV–vis *Jasco* V-750 Spectrometer, with a standard 10 mm light pass *Hellma* Semi-Micro cuvette (114-10-40) at 25 ºC, using the following settings: UV-vis bandwidth 5.0 nm, UV-vis response 0.06 s; data interval 0.5 nm; scan mode continuous, scan speed 200 nm/min. 5579 $M^{-1}cm^{-1}$ at 278 nm in 0.1 M phosphate buffer, pH 7.0 was used as molar extinction coefficient for Trp[1] and 10964 $M^{-1}cm^{-1}$ at 344 nm in 10 mM HEPES, pH 8.0 was used as molar extinction coefficient for 1,8-naphthalimide.[2]

Luminescence experiments were carried out as described below:

Setup 1: Time-gated emission measurements were performed with a FluoroMax Plus-P Spectrofluorometer from *Horiba Scientific* in the phosphorescence mode using a xenon flash lamp, equipped with an R928P photon counting emission detector and a Peltier temperature control system (water cooled) from *Quantum Northwest*. The measurements were carried out with the following settings: excitation wavelength 282 nm or 344 nm for **P1[Tb]** or **P2[Eu]** complexes, respectively; excitation slit width 10.0 nm, emission slit width 3.0 nm; increment 1.0 nm; time between flashes 0.061 s; initial delay 0.2 ms; sample window 0.02 s; flash count 0.01 s; HV detector voltage 950 V. Some measurements were also performed with an *Agilent* Cary Eclipse Fluorescence Spectrophotometer in the phosphorescence mode, equipped with a Peltier temperature

control system (water cooled). The measurements were carried out with the following settings: excitation wavelength 282 nm or 344 nm for **P1[Tb]** or **P2[Eu]** complexes, respectively; excitation slit width 5.0 nm, emission slit width 2.5 nm; increment 1.0 nm; averaging time 0.1 s; gate time 5 ms; total decay time 0.02 s; delay time 0.2 ms; PMT detector voltage 1000 V.

Setup 2: Steady-state emission measurements were performed with a FluoroMax Plus-P Spectrofluorometer from *Horiba Scientific,* equipped with a Peltier temperature control system (water cooled) from *Quantum Northwest*. The measurements were carried out with the following settings: excitation wavelength 282 nm or 344 nm for **P1[Tb]** or **P2[Eu]** complexes, respectively; excitation slit width 10.0 nm, emission slit width 3.0 nm; increment 1.0 nm; 2 averaged scans. A 475 nm long-pass filter was used to avoid the interference of harmonic doubling.

All measurements were carried out with a *Hellma* Semi-Micro cuvette (114F-10-40) or a *Starna Scientific* Magnetic Stirring Micro cuvette (28-F/Q/10). The emission spectra for Tb(III) and Eu(III) complexes were recorded from 450 to 600 nm and 550 to 750 nm, respectively.

Bacterial strains and culture conditions

**Bacterial Strains**	**Description**	**Reference/Origin**
P. aeruginosa PA14	Wild-type PA14 Strain	3
P. aeruginosa PA14 Tn-lasB-1	Tn insertion position within *lasB* (bp) 1120. Position in PA14 transposon mutant library: PAMr_nr_mas_05_2:F6	4
P. aeruginosa PA14 Tn-lasB-2	Tn insertion position within *lasB* (bp) 893. Position in PA14 transposon mutant library: PAMr_nr_mas_11_4:A5	4
E. coli	NEB Stable Competent *E. coli*	New England Biolabs (#C3040I)

Table S1: *P. aeruginosa* and *E. coli* strains used in this work**.**

Bacterial samples were grown overnight in 10 mL of LB medium at 37 °C. After incubation, samples were collected and spun down by centrifugation (7000 rpm, 10 min), and the supernatants were filtered through 0.2 µm pore-size filters (Whatman).

**Bacterial Strains**	**CFU/mL**	**Elastase production (O.D.$_{690}$)**
P. aeruginosa PA14	$(4.4 \pm 1)\times10^9$	0.905 ± 0.007
P. aeruginosa LasB1 mutant	$(4.9 \pm 0.5)\times10^9$	0.00 ± 0.00
P. aeruginosa LasB2 mutant	$(4.7 \pm 0.3)\times10^9$	0.00 ± 0.00
E. coli	1.45×10^9	-

Table S2: Growth characteristics and elastase production by the *P. aeruginosa* strains

Peptide synthesis

Peptides were synthesized using standard Fmoc/*t*Bu solid phase peptide synthesis procedures. Amino acid couplings were conducted using a 4-fold excess and HBTU (4 equiv.) as activating agent. Each amino acid was activated for 2 min in DIEA/DMF (6 equiv.) before being added onto the resin. Peptide bond-forming couplings were carried out for 30 min.

P1[Tb] metallopeptide

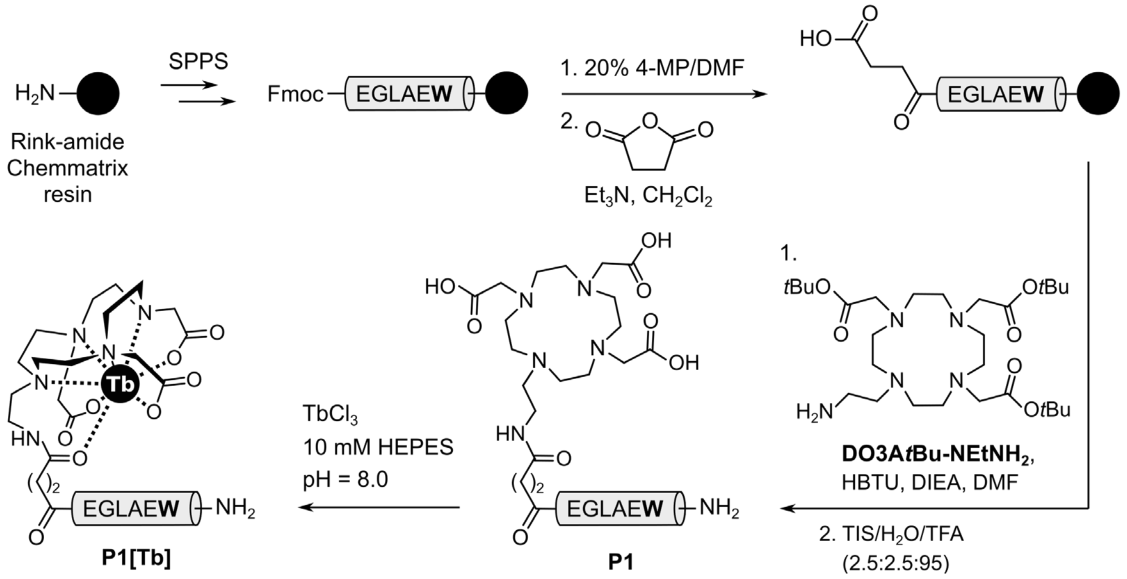

Scheme S1. Synthetic scheme of the metallopeptide **P1[Tb]**.

Succinylation of the *N*-terminus: After the deprotection of the Fmoc group by treating the resin (0.1 mmol) with a 20% 4-methylpiperidine (4-MP) solution in DMF for 15 min (5 mL), the *N*-terminus of the peptide was succinylated by treatment of the resin with a mixture of succinic anhydride (10 equiv.) and Et_3N (1.1 equiv.) in CH_2Cl_2 (5 mL) overnight. After filtration, the resin was washed with CH_2Cl_2 (3 × 5 mL × 3 min).

DO3A*t*Bu-NEtNH$_2$ coupling: DO3A*t*Bu-NEtNH$_2$ was synthesized following published procedures.[5,6] A solution of HBTU (1 equiv.) in DMF (1 mL) was mixed with DIEA/DMF (0.195 M, 4 equiv., 2 mL) and the mixture was added to the resin (0.06 mmol) and mixed for 2 min before adding a solution of DO3A*t*Bu-NEtNH$_2$ (2 equiv.) in DMF (2 mL) over the resin suspension. The mixture was stirred under N_2 for 2 h. After filtration, the resin was washed with DMF (3 × 3 mL × 3 min) and CH_2Cl_2 (3 × 3 mL × 3 min).

Cleavage and deprotection of semipermanent protecting groups: The cleavage/deprotection step was performed adding a TFA cleavage cocktail (2.5% H_2O, 2.5% triisopropysilane (TIS) and 95% TFA) to the resin-bound peptides, and the mixture was shaken for 4.5 h. After precipitation of the TFA filtrate in cold Et_2O, the peptide was dissolved in 1:1 MeCN/H_2O. The purification of the peptide was performed in a *JASCO* LC-4000 series using an Aeris semipreparative column from *Phenomenex* (peptide XB-C18 stationary phase, 5 µm, 100 Å pore size, 250 × 10 mm). The method used for semipreparative HPLC was

1 → 10% B over 23 min. Solvent A: 20 mM NH_4Ac, solvent B: 1% NH_4Ac (20 mM), 99% MeCN. The collected fractions were lyophilized and stored at −20 °C.

P1: HPLC: t_R = 13.9 min. ESI-MS (m/z): $[MH]^+$ calculated for $C_{52}H_{79}N_{13}O_{18}$: 1174.5739; found 1174.5749 $[M+H]^+$, 1196.5565 $[M+Na]^+$, 587.7910 $[M+2H]^{2+}$.

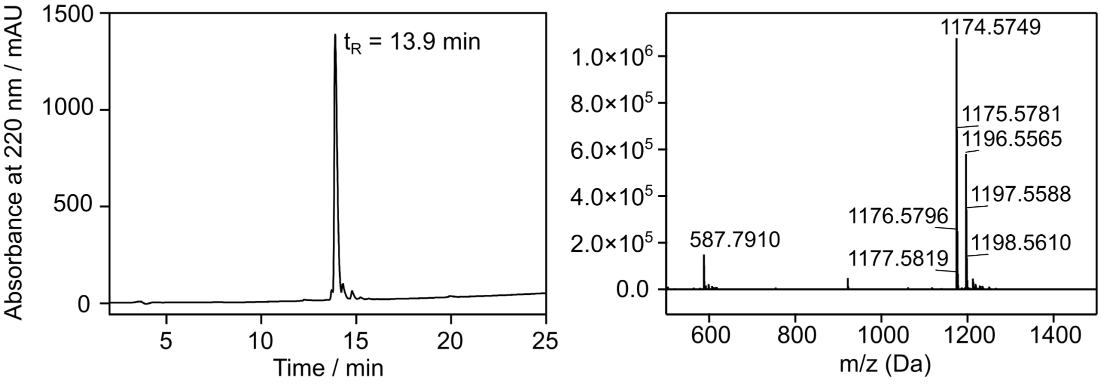

Figure S1. HPLC chromatogram at 220 nm (left) and ESI-MS spectrum of the t_R = 13.9 min peak, identified as **P1** (right).

Tb(III) chelation: 1.4 μL of a 10 mM $TbCl_3$ solution in 1 mM HCl (1 equiv.) were added to a 0.7 mM **P1** solution in HEPES buffer (10 mM HEPES, pH 8, 20 μL) and the mixture was shaken for 30 min and analyzed by reversed-phase HPLC-MS.

P1[Tb]: ESI-MS (m/z): $[MH]^+$ calculated for $C_{52}H_{76}N_{13}O_{18}Tb$: 1330.4758; found 665.7411 $[M+2H]^{2+}$.

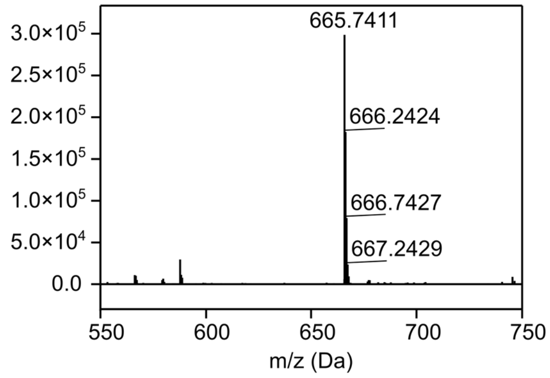

Figure S2. ESI-MS spectrum of the **P1[Tb]** solution.

P2[Eu] metallopeptide

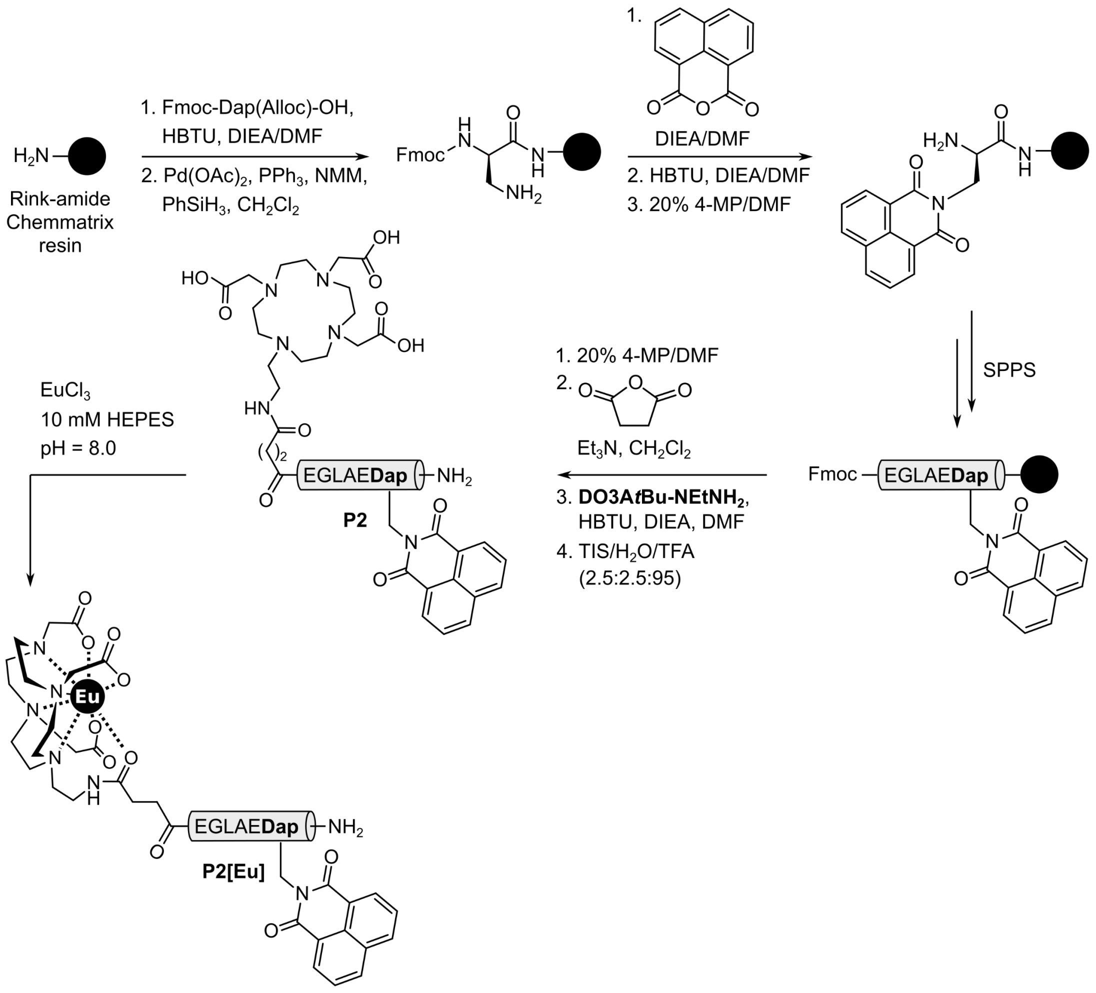

Scheme S2. Synthetic scheme of the metallopeptide **P2[Eu]**.

Fmoc-Dap(Alloc)-OH coupling and orthogonal side chain deprotection: Fmoc-Dap(Alloc)-OH (4 equiv.) was manually coupled to the resin (0.1 mmol) following standard Fmoc solid phase peptide synthesis. The side chain of the Dap residue was selectively deprotected by treating the resin (0.1 mmol) with a mixture of $Pd(OAc)_2$ (0.3 equiv.), PPh_3 (1.5 equiv.), *N*-methylmorpholine (NMM, 10 equiv.), and $PhSiH_3$ (10 equiv.) in CH_2Cl_2 (5 mL) overnight at RT. The resin was then filtered and washed with DMF (3 × 5 mL × 3 min), *N,N*-diethyldithiocarbamate (DEDTC) (25 mg in 5 mL of DMF, 10 min), CH_2Cl_2 (3 × 5 mL × 3 min) and dried under N_2 (g).

1,8-Naphthalic anhydride coupling to the Dap side chain: 1,8-Naphthalic anhydride was coupled to the side chain of the Dap residue attached to the resin using a double coupling procedure as reported in literature.[7] A solution of 1,8-naphthalic anhydride (0.2 mmol, 2 equiv.) was prepared in 3 mL DIEA/DMF (0.195 M, 0.6 mmol, 6 equiv.) and the resulting mixture was added over the resin (0.1 mmol). The resin suspension was stirred overnight and was then filtered and washed with DMF (3 × 5 mL × 3 min). The antenna was further cycled by adding a mixture of HBTU (0.2 mmol, 2 equiv.) and DIEA (0.6 mmol, 6 equiv.) in DMF (5 mL) over the resin (0.1 mmol). N_2 was bubbled through the resin suspension for 45 min

and the resin was then filtered and washed with DMF (2 × 5 mL × 3 min). The *N*-terminal Fmoc group was removed by treating the resin (0.1 mmol) with a 20% 4-MP solution in DMF (5 mL) for 15 min.

Succinylation of the *N*-terminus: The *N*-terminus of the peptide was succinylated by treatment of the resin (0.1 mmol) with a mixture of succinic anhydride and Et_3N in CH_2Cl_2, following the same protocol previously described for **P1**.

DO3A*t*Bu-NEtNH$_2$ coupling: The chelating macrocyclic **DO3A*t*Bu-NEtNH$_2$** was coupled to the peptide in solid phase (0.1 mmol) following the same protocol described above for **P1**.

Cleavage and deprotection of semipermanent protecting groups: The cleavage/deprotection step was performed adding the TFA cleavage cocktail to the resin-bound peptide, and the mixture was shaken for 6 h. After precipitation of the TFA filtrate in cold Et_2O, the peptide was dissolved in 1:1 MeCN/H_2O. The purification of the peptide was performed in an *Agilent* 1200 series using an Aeris semipreparative column from Phenomenex (peptide XB-C18 stationary phase, 5 µm, 100 Å pore size, 250 × 10 mm). The method used for semipreparative HPLC was 15 → 46 % MeCN, 0.1% TFA / H_2O, 0.1% TFA over 6 min and then a 46 → 47 % MeCN, 0.1% TFA / H_2O, 0.1% TFA gradient over 15 min. The collected fractions were lyophilized and stored at −20 °C.

P2: HPLC: t_R =14.5 min. ESI-MS (m/z): $[MH]^+$ calculated for $C_{56}H_{79}N_{13}O_{20}$: 1254.5638; found 1254.5634 $[M+H]^+$, 627.7859 $[M+2H]^{2+}$.

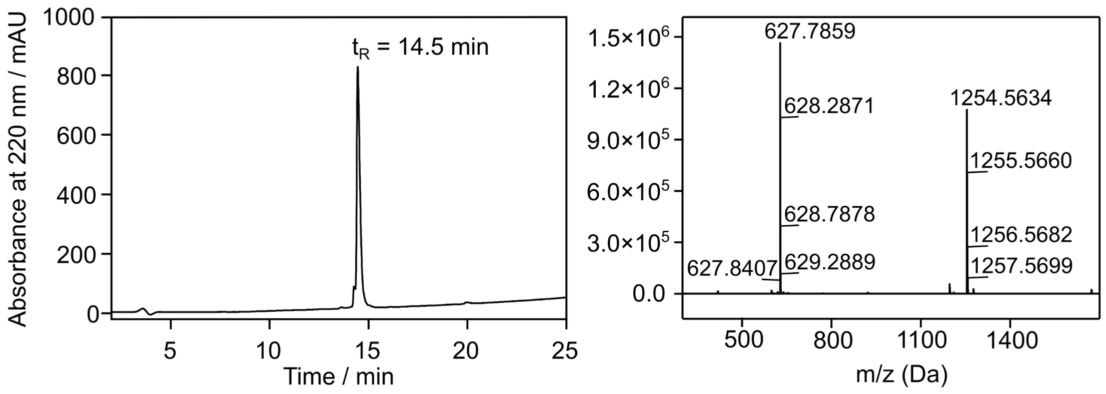

Figure S3. HPLC chromatogram at 220 nm (left) and ESI-MS spectrum of the t_R = 14.5 min peak, identified as **P2** (right).

Eu(III) chelation: 2 µL of a 2.5 mM $EuCl_3$ solution in 1 mM HCl (1 equiv.) were added to a 50 µM **P2** solution in HEPES buffer (10 mM HEPES, pH 8, 100 µL) and the mixture was shaken for 30 min and analyzed by reversed-phase HPLC-MS.

P2[Eu]: ESI-MS (m/z): $[MH]^+$ calculated for $C_{56}H_{76}EuN_{13}O_{20}$: 1404.4615; found 1404.4636 $[M+H]^+$, 702.7357 $[M+2H]^{2+}$.

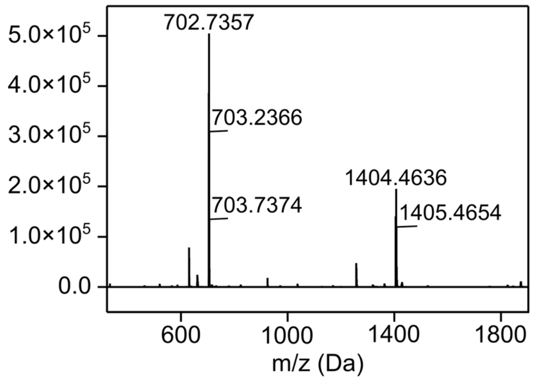

Figure S4. ESI-MS spectrum of the **P2[Eu]** solution.

Luminescent spectra of P1[Tb] and P2[Eu]

Time-gated emission titrations: To 1 mL of a 15 µM **P1** or **P2** solution in HEPES buffer, aliquots of a 2.5 mM stock solution of $TbCl_3$ or $EuCl_3$ in 1 mM HCl were added at 25 ºC. The luminescence spectra were recorded upon each addition using *setup 1* parameters.

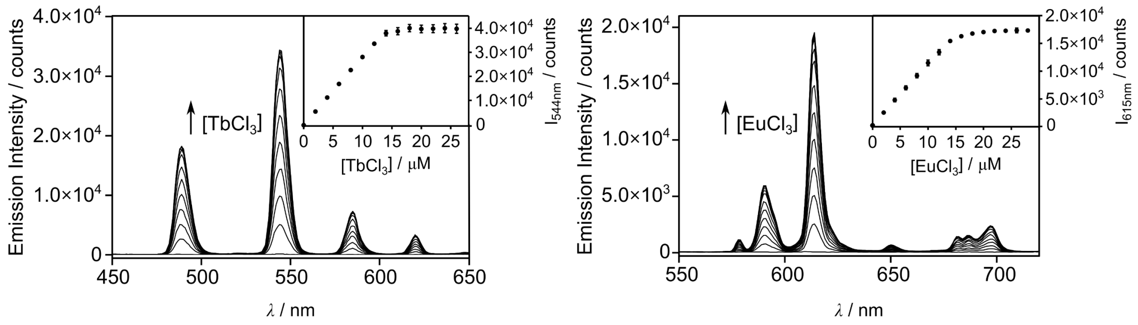

Figure S5. Luminescent spectra recorded at 25 ºC of a 15 µM **P1** solution in HEPES buffer with increasing concentrations of $TbCl_3$ (left) and a 15 µM **P2** solution in HEPES buffer with increasing amounts of $EuCl_3$ (right). Inset: emission at 544 nm (left) or 615 nm (right) vs the concentration of the metal ions.

Time course experiments of P1[Tb] with LasB

To a 10 µM **P1[Tb]** solution in HEPES buffer (1 mL) 1 µL of a 1 M $CaCl_2$ solution (100 equiv.) was added prior to the addition of 1 µL of a 2 mg/mL LasB solution in milli-Q water (final concentration 2 µg/mL, 60.6 nM, 0.006 equiv.). Time-gated emission spectra were recorded every hour for 15 h at 37 ºC with magnetic stirring at 70 rpm using *setup 1* parameters.

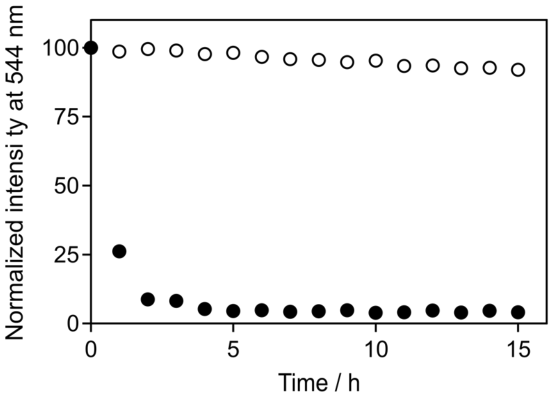

Figure S6. Time courses of 10 µM **P1[Tb]** and 1 mM $CaCl_2$ in HEPES buffer at 37 ºC in the absence (○) and in the presence (●) of 2 µg/mL LasB.

Luminescence spectra of P1[Tb] in the presence of human leukocyte elastase (HLE) and trypsin

To 1 mL of a 5 µM **P1[Tb]** solution in HEPES buffer, 0.5 µL of a 67.8 µM HLE solution in 50 mM NaOAc, 200 mM NaCl, pH 5.5 (final concentration 1 µg/mL, 33.9 nM, 0.0068 equiv.) were added. The final mixture was stirred at 37 ºC and 70 rpm for 20 h at RT. Time-gated emission spectra were recorded at 37 ºC immediately and 20 h after enzyme addition using *setup 1* parameters.

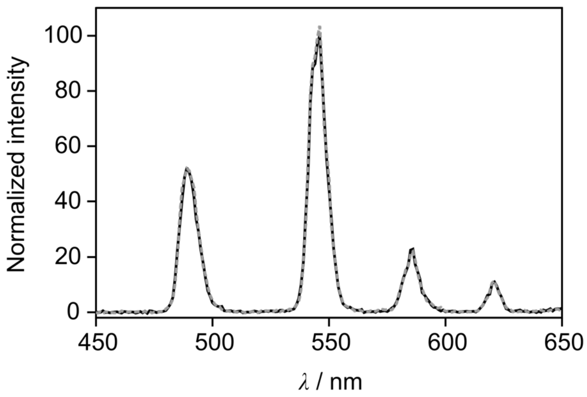

Figure S7. Time-gated emission spectra recorded at 37 ºC of a 5 µM **P1[Tb]** solution in HEPES buffer immediately (—) and 20 h after the addition (---) of HLE (1 µg/mL final concentration).

To 1 mL of a 10 µM solution of **P1[Tb]** and 1 mM $CaCl_2$ (100 equiv.) in HEPES buffer, 3.5 µL of a 41 µM solution of trypsin (3.5 µg/mL final concentration, 0.014 equiv.) were added. The final solution was stirred at 70 rpm for 20 h at RT. Time-gated emission spectra were recorded at 25 ºC immediately and 20 h after enzyme addition using *setup 1* parameters.

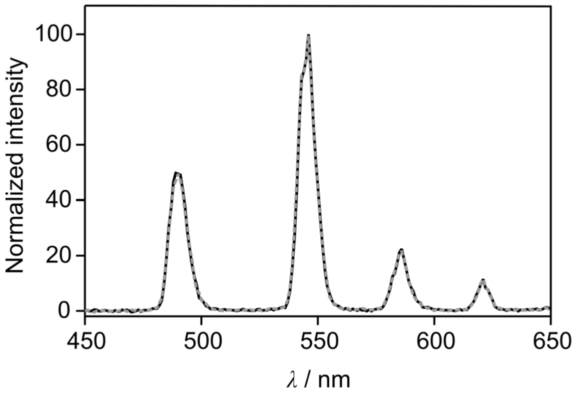

Figure S8. Time-gated emission spectra recorded at 25 ºC of a 10 µM **P1[Tb]** and 1 mM $CaCl_2$ solution in HEPES buffer immediately (—) and 20 h after the addition (---) of trypsin (3.5 µg/mL final concentration).

Determination of P1[Tb] specificity constant (k_{sub}) for LasB

To 1 mL of 10, 7.5, 5, and 1 µM solutions of **P1[Tb]** and 1 mM $CaCl_2$ in HEPES buffer, 1 µL of a 2 mg/mL solution of LasB in water was added (final concentration 2 µg/mL, 60.6 nM). Time-gated emission spectra were recorded every 15 min for 10 h at 37 ºC with magnetic stirring at 70 rpm using *setup 1* parameters.

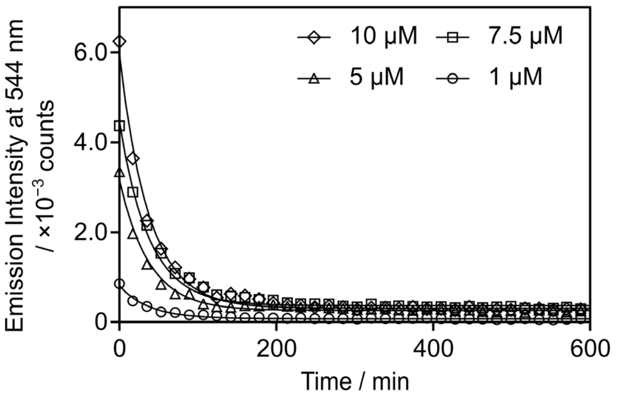

Figure S9. Time courses for 1 µM (○), 5 µM (△), 7.5 µM (□) and 10 µM (◇) **P1[Tb]** and 1 mM $CaCl_2$ solutions in HEPES buffer (10 mM HEPES, pH 8.0) with 2 µg/mL (60.6 nM) LasB at 37 ºC.

The obtained data were fitted to the "hit-and-run" model,[8] using the *Dynafit* software.[9]

$$\textbf{E + P1[Tb]} \xrightarrow{k_{sub}} \textbf{E +} \text{F1[Tb] + F2}$$

Scheme S3. Scheme of the "hit-and-run" model for the reaction of the metallopeptide **P1[Tb]** in the presence of LasB (E), giving the two peptide fragments (**F1[Tb]** and **F2)** as products.

Determination of the LOD and LOQ with P1[Tb]

Calibration curves: The time-gated emission at 544 nm of solutions containing 1, 5, or 10 µM **P1[Tb]**, and 1 mM $CaCl_2$ in HEPES buffer, was recorded at 37 ºC using *setup 1*. For the cleaved probe, solutions containing 1, 5, or 10 µM **P1[Tb]** and 1 mM $CaCl_2$ in HEPES buffer were incubated with 1 µL of a 2 mg/mL LasB solution (2 µg/mL final concentration, 0.00606 equiv.) for 8 h at 100 rpm and 37 ºC. After this time, the time-gated emission at 544 nm was recorded at 37 ºC using *setup 1*.

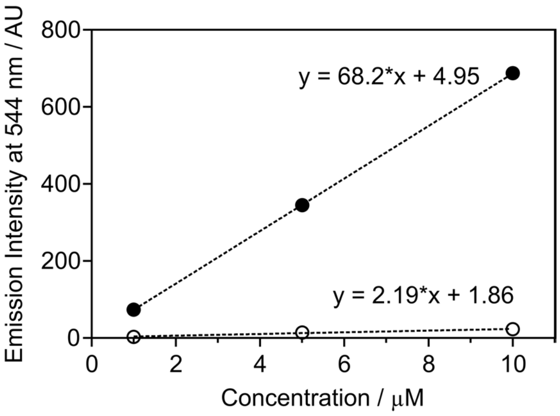

Figure S10. Emission intensity at 544 nm vs the concentration of uncleaved (●) and cleaved **P1[Tb]** (○) and the best fit.

LOD and LOQ determination: 5 µM **P1[Tb]** and 500 µM $CaCl_2$ solutions in HEPES buffer were incubated with LasB (final concentrations: 100, 60, 30, 10, 5, 1 nM). The resulting solutions were stirred for 1 h at 100 rpm and 37 ºC, and their time-gated emission spectra were then recorded at 37 ºC using *setup 1*. The luminescence calibration curves were used to convert the luminescence values (in arbitrary units) to molar concentrations of cleaved **P1[Tb]** (in µM). The results were then fitted to a sigmoidal four-parameter logistic (4PL) curve.[10]

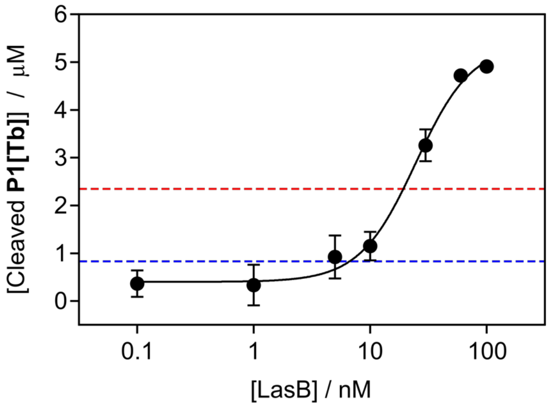

Figure S11. Cleaved **P1[Tb]** concentration after 1 h vs LasB concentration. Detection (---) and quantification (---) thresholds are indicated with dashed lines.

The LOQ and LOD were quantified as the target concentration required to achieve the end-point quantification and detection thresholds, $C_{Q,NC} = \mu_{NC} + 10\sigma_{NC}$ and $C_{D,NC} = \mu_{NC} + 3\sigma_{NC}$, respectively. Here, μ_{NC} and σ_{NC} represent the mean and standard deviation of the concentration of the non-cleaved complex in absence of LasB.[11]

Steady-state vs time-gated luminescence spectra of P1[Tb] in the presence of the supernatant from a LasB-deficient *P. aeruginosa* strain culture

To 790 µL of a 10 µM solution of **P1[Tb]** and 1 mM $CaCl_2$ (100 equiv.) in HEPES buffer, 10 µL of the *P. aeruginosa* LasB2 mutant strain supernatant were added. Time-gated and steady-state emission measurements were recorded at 37 ºC using *setup 1* and *2* parameters, respectively.

Time course experiments of P1[Tb] metallopeptide with *P. aeruginosa* supernatants

To 790 µL of a 10 µM solution of **P1[Tb]** and 1 mM $CaCl_2$ (100 equiv.) in HEPES buffer, 10 µL of supernatant from cultures of a *P. aeruginosa* PA14 strain producing LasB or *P. aeruginosa* mutant strains deficient in LasB (LasB1 or LasB2) were added. Time-gated emission spectra were recorded every hour for 12 h at 37 ºC with magnetic stirring at 70 rpm using *setup 1* parameters.

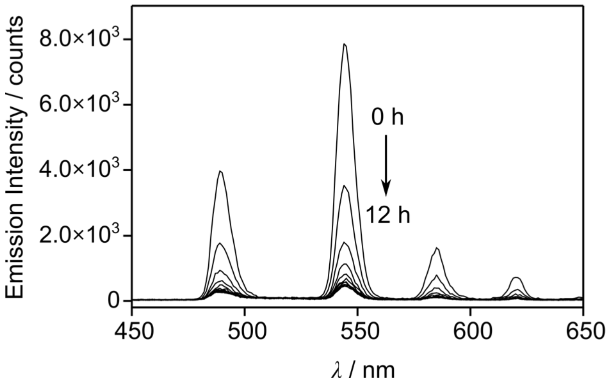

Figure S12. Luminescence spectra recorded at 37 ºC of 10 µM **P1[Tb]** and 1 mM $CaCl_2$ in HEPES buffer in the presence of 10 µL of *P. aeruginosa* PA14 supernatant up to 12 hours after the addition.

The mixtures of **P1[Tb]** in the presence of supernatant from cultures of *P. aeruginosa* strains, producing LasB or the LasB-deficient mutants, were analyzed by HPLC-MS. To do so, 9 µL of the corresponding supernatants were added to a 0.5 mM **P1[Tb]** and 50 mM $CaCl_2$ solution in HEPES buffer. The solutions were kept at RT for 1 h before injection. Based on the preferential cleavage sequence of LasB, we expected **P1[Tb]** cleavage to produce the following fragments: **DO3A[Tb]-NEtNH$_2$-Suc-EG-OH** (**F1[Tb]**) and **H-LAEW-NH$_2$** (**F2**) (Scheme S4).

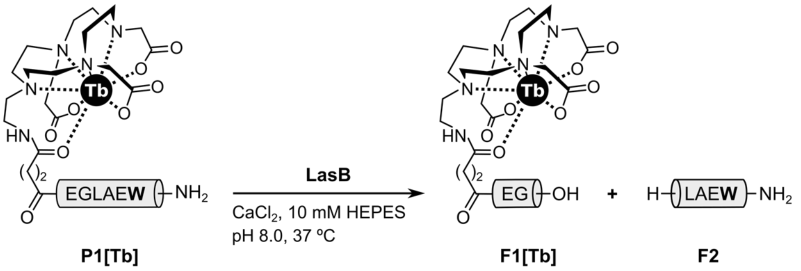

Scheme S4. Schematic representation of the **P1[Tb]** cleavage by the action of LasB, generating fragments **F1[Tb]** and **F2**.

F1[Tb]: HPLC: t_R =10.9 min. ESI-MS (m/z): $[MH]^+$ calculated for $C_{27}H_{42}N_7O_{13}Tb$: 832.21; found 832.21 $[M+H]^+$, 416.61 $[M+2H]^{2+}$.

F2: ESI-MS (m/z): $[MH]^+$ calculated for $C_{25}H_{36}N_6O_6$: 517.28; found 517.27 $[M+H]^+$.

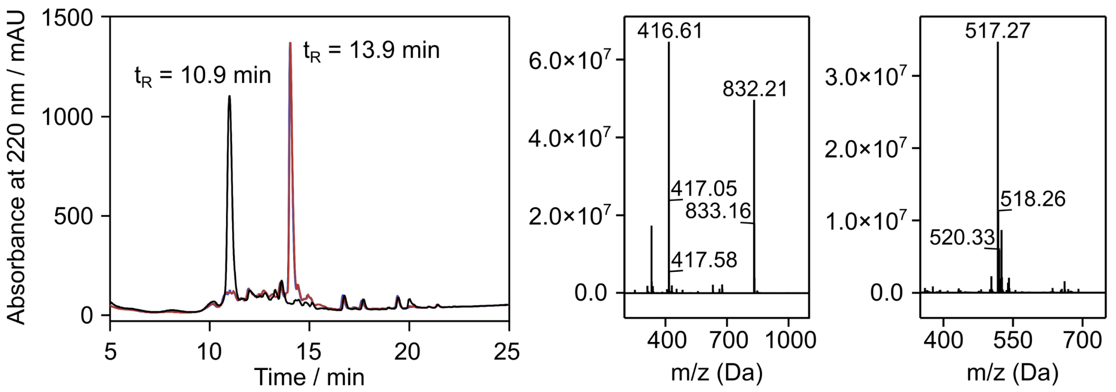

Figure S13. Overlapped HPLC chromatograms at 220 nm of **P1[Tb]** and $CaCl_2$ solutions in the presence of the *P. aeruginosa* supernatants from the wild-type (—), LasB1 mutant (—), and LasB2 mutant (—) strains (left), being the peak at t_R = 13.9 min **P1[Tb]**. ESI-MS spectra of the t_R = 10.9 min peak identified as **F1[Tb]** (centre) and of **F2** (right), identified at t_R = 9.0 min in the total ion chromatogram (TIC).

Luminescence spectra of P1[Tb] in the presence of an *E. coli* supernatant

To 1 mL of a 10 µM solution of **P1[Tb]** and 1 mM $CaCl_2$ (100 equiv.) in HEPES buffer, 10 µL of supernatant from an *E. coli* culture were added. The final solution was stirred at 70 rpm for 20 h at 37 ºC. Time-gated emission spectra were recorded at 37 ºC immediately and 20 h after the supernatant addition using *setup 1* parameters.

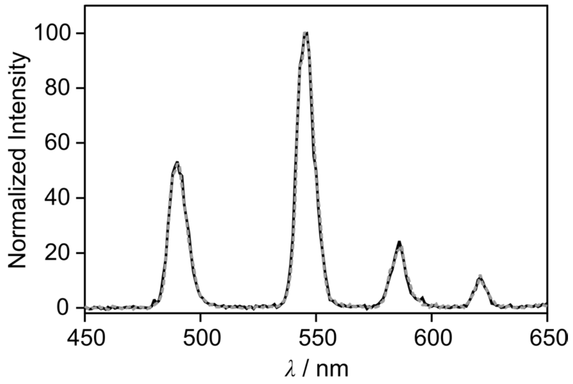

Figure S14. Time-gated emission spectra recorded at 37 ºC of a 10 µM **P1[Tb]** and 1 mM $CaCl_2$ solution in HEPES buffer immediately (—) and 20 h after the addition (---) of an *E. coli* supernatant.

Luminescence spectra of P1[Tb] in the presence of the *P. aeruginosa* supernatant secreting LasB and EDTA

To 1 mL of a 5 µM solution of **P1[Tb]**, 0.5 mM $CaCl_2$ (100 equiv.), and 5 mM EDTA (1000 equiv.) in HEPES buffer, 6.5 µL of the supernatant from the *P. aeruginosa* strain producing LasB were added.

Control solutions containing 5 µM of **P1[Tb]**, 0.5 mM $CaCl_2$ (100 equiv.), and 5 mM EDTA (1000 equiv.) in HEPES buffer or 5 µM of **P1[Tb]**, 0.5 mM $CaCl_2$ (100 equiv.) in HEPES buffer and 6.5 µL of the supernatant from the *P. aeruginosa* strain producing LasB, were also prepared.

All three solutions were incubated at 37 ºC with magnetic stirring at 70 rpm for 3 h. After incubation, the time-gated emission spectra were recorded at 37 ºC using *setup 1* parameters.

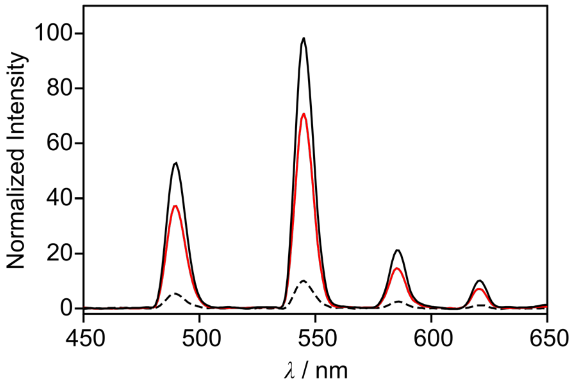

Figure S15. Time-gated emission spectra recorded at 37 ºC of a 5 μM **P1[Tb]** and 0.5 mM $CaCl_2$ solution in HEPES buffer containing (—) 5 mM EDTA, (—) 5 mM EDTA and 6.5 μL of the *P. aeruginosa* supernatant, or (---) 6.5 μL of the *P. aeruginosa* supernatant.

Luminescence spectra of P1[Tb] in the presence of glutathione (GSH) and H_2O_2

To 900 μL of a 5.5 μM solution of **P1[Tb]** in HEPES buffer, 100 μL of a 1 mM solution of GSH in water (final concentration 100 μM, 20 equiv.) were added. Time-gated emission spectra were recorded at 37 ºC before and 20 h after the addition using *setup 1* parameters.

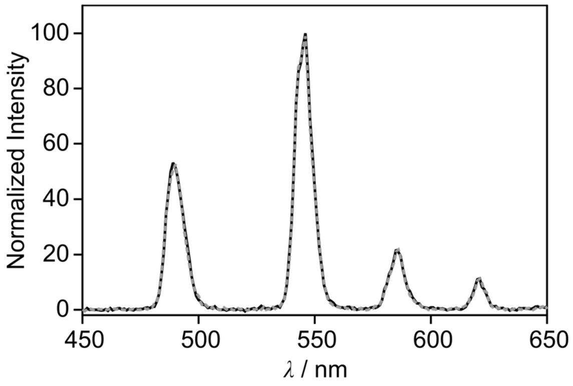

Figure S16. Time-gated emission spectra recorded at 37 ºC of a 5 μM **P1[Tb]** solution in HEPES buffer before (—) and 20 h after the addition (---) of GSH.

To 1 mL of a 5 μM solution of **P1[Tb]** in HEPES buffer, 1 μL of a 500 mM solution of H_2O_2 in water (final concentration 0.5 mM, 100 equiv.) were added. Time-gated emission spectra were recorded at 37 ºC before and 20 h after the addition using *setup 1* parameters.

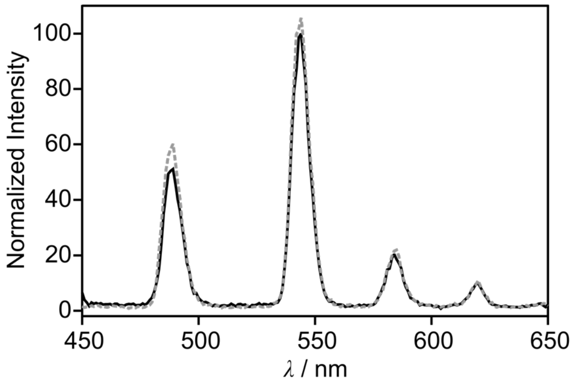

Figure S17. Time-gated emission spectra recorded at 37 ºC of a 5 μM **P1[Tb]** solution in HEPES buffer before (—) and 20 h after the addition (---) of H_2O_2.

Luminescence spectra of P1[Tb] in the presence of pyocyanin and *P. aeruginosa* supernatant

To 1 mL of a 5 μM solution of **P1[Tb]** in HEPES buffer, 1 μL of a 1 mM solution of pyocyanin in ethanol (final concentration 1 μM, 0.2 equiv.) were added. Time-gated emission spectra were recorded at 37 ºC before, immediately and 20 h after the addition using *setup 1* parameters.

To a solution containing 5 μM solution of **P1[Tb]** and 1 μM pyocyanin in HEPES buffer, 0.5 μL of a 1 M $CaCl_2$ solution and 6.5 μL of the supernatant from the *P. aeruginosa* strain culture producing LasB were added. Time-gated emission spectra of the resulting mixture were recorded every hour for 20 h at 37 ºC with magnetic stirring at 70 rpm using *setup 1* parameters.

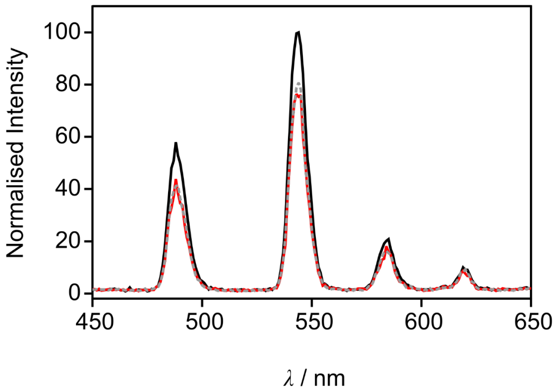

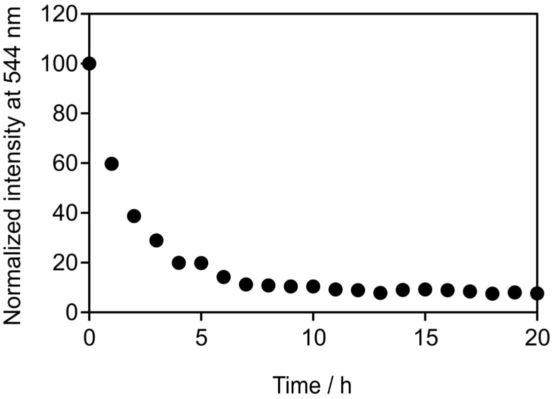

Figure S18. Time-gated emission spectra recorded at 37 ºC of a 5 μM **P1[Tb]** solution in HEPES buffer before (—), immediately (—), and 20 h after (---) the addition of pyocyanin (left). Time course of a solution containing 5 μM **P1[Tb]**, 1 μM of pyocyanin, 0.5 mM $CaCl_2$, and the supernatant from the *P. aeruginosa* strain producing LasB at 37 ºC (right).

Luminescence spectra of P1[Tb] in the presence of 1.2% FBS and *P. aeruginosa* supernatant

To 987 μL of a 10 μM **P1[Tb]** solution in HEPES buffer, 12.5 μL of FBS (final concentration 1.2% v/v) were added. Time-gated emission spectra of the resulting mixture were recorded every hour for 12 h at 37

ºC with magnetic stirring at 70 rpm using *setup 1* parameters. Subsequently, 1 µL of a 1 M $CaCl_2$ solution and 12.5 µL of the supernatant from the *P. aeruginosa* strain culture producing LasB were added. Time-gated emission spectra of the resulting mixture were recorded every hour for 12 h at 37 ºC with magnetic stirring at 70 rpm using *setup 1* parameters.

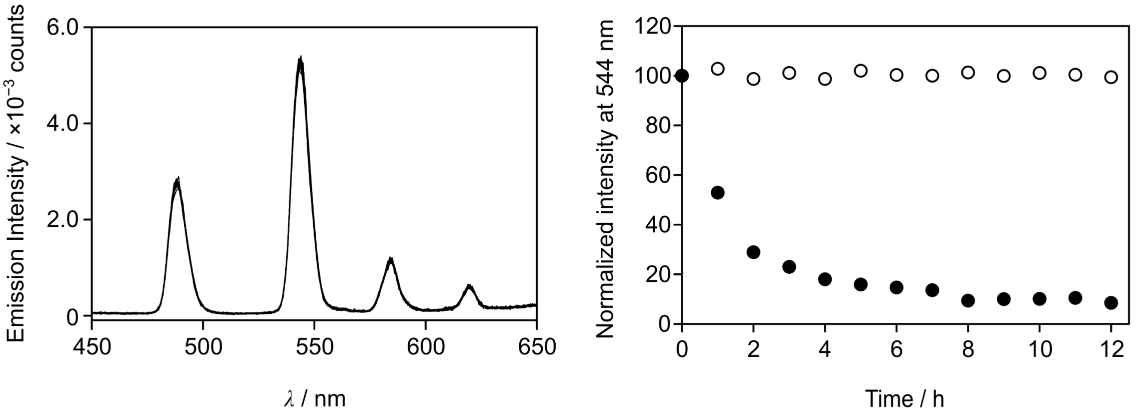

Figure S19. Luminescence spectra of 10 µM **P1[Tb]** in HEPES buffer with 1.2% v/v FBS recorded over time at 37 ºC up to 12 h after the addition of FBS (left). Time courses of 10 µM **P1[Tb]** and 1.2% v/v FBS (○), and 10 µM **P1[Tb]**, 1 mM $CaCl_2$, 1.2% v/v FBS and the supernatant from the *P. aeruginosa* strain producing LasB (●) at 37 ºC (right).

Determination of P2[Eu] specificity constant (k_{sub}) for LasB

To 1 mL of 10, 7.5, 5, and 1 µM solutions of **P2[Eu]** and 1 mM $CaCl_2$ in HEPES buffer, 1 µL of a 2 mg/mL solution of LasB in water was added (final concentration 2 µg/mL, 60.6 nM). Time-gated emission spectra were recorded every 15 min for 10 h at 37 ºC with magnetic stirring at 70 rpm using *setup 1* parameters.

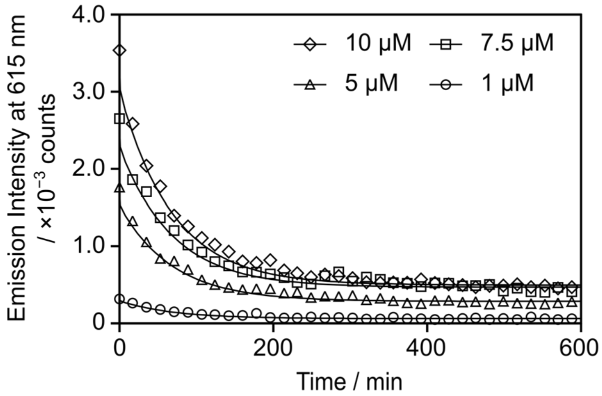

Figure S20. Time courses for 1 µM (○), 5 µM (△), 7.5 µM (□) and 10 µM (◇) **P2[Eu]** and 1 mM $CaCl_2$ solutions in HEPES buffer (10 mM HEPES, pH 8.0) with 2 µg/mL (60.6 nM) LasB at 37 ºC.

The obtained data were fitted to the "hit-and-run" model,[8] using the *Dynafit* software.[9]

$$\mathbf{E + P2[Eu]} \xrightarrow{k_{sub}} \mathbf{E +} \text{ F1[Eu] + F3}$$

Scheme S5. Scheme of the "hit-and-run" model for the reaction of the metallopeptide **P2[Eu]** in the presence of LasB (E), giving the two peptide fragments (**F1[Tb]** and **F3)** as products.

Determination of the LOD and LOQ with P2[Eu]

Calibration curve: The time-gated emission at 615 nm of solutions containing 1, 5, or 10 µM **P2[Eu]**, and 1 mM $CaCl_2$ in HEPES buffer, was recorded at 37 ºC using *setup 1*. For the cleaved probe, solutions containing 1, 5, or 10 µM **P2[Eu]** and 1 mM $CaCl_2$ in HEPES buffer were incubated with 1 µL of a 2 mg/mL LasB solution (2 µg/mL final concentration, 0.00606 equiv.) for 8 h at 100 rpm and 37 ºC. After this time, the time-gated emission at 615 nm was recorded at 37 ºC using *setup 1*.

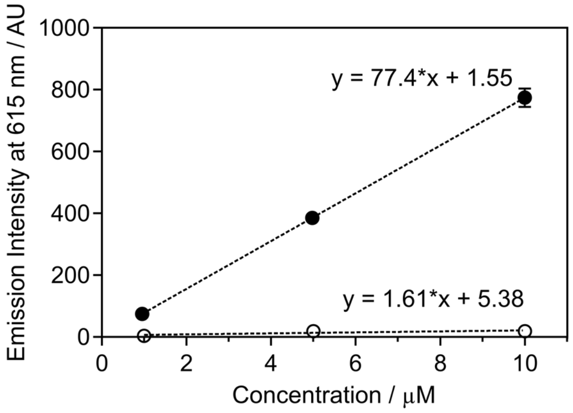

Figure S21. Emission intensity at 615 nm vs the concentration of uncleaved (●) and cleaved **P2[Eu]** (○) and the best fit.

LOD and LOQ determination: 5 µM of **P2[Eu]** and 500 µM of $CaCl_2$ solutions in HEPES buffer were incubated with LasB (final concentrations: 100, 60, 30, 10, 5, 1 nM). The resulting solutions were stirred for 1 h at 100 rpm and 37 ºC, and their time-gated emission spectra were then recorded at 37 ºC using *setup 1*. The luminescence calibration curves were used to convert the luminescence values (in arbitrary units) to molar concentrations of cleaved **P2[Eu]** (in µM). The results were then fit to a sigmoidal four-parameter logistic (4PL) curve.[10] The LOQ and LOD were then quantified using the same method as that used for **P1[Tb]**.

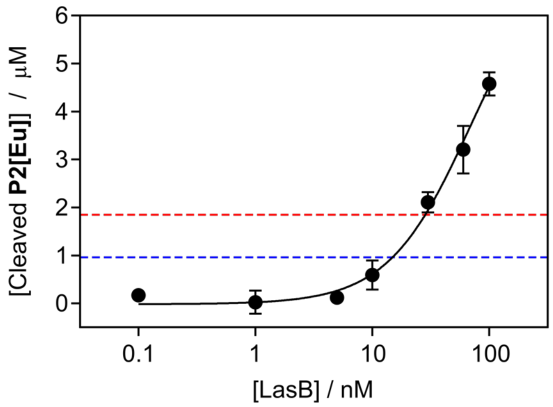

Figure S22. Cleaved **P2[Eu]** concentration after 1 h vs LasB concentration. Detection (---) and quantification (---) thresholds are indicated with dashed lines.

Time course experiments of P2[Eu] metallopeptide with *P. aeruginosa* supernatants

To 790 µL of a 10 µM solution of **P2[Eu]** and 1 mM $CaCl_2$ (100 equiv.) in HEPES buffer, 10 µL of the supernatants from cultures of *P. aeruginosa* strains, producing the LasB protease or the LasB-deficient mutants, were added. Time-gated emission spectra were recorded every hour for 12 h at 37 ºC with magnetic stirring at 70 rpm using *setup 1* parameters.

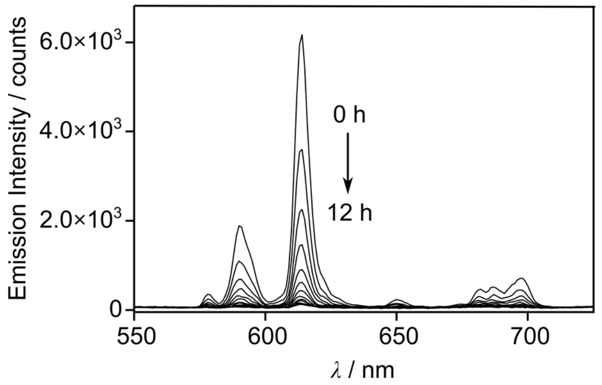

Figure S23. Luminescence spectra recorded at 37 ºC of 10 µM **P2[Eu]** and 1 mM $CaCl_2$ in HEPES buffer in the presence of 10 µL of wild-type *P. aeruginosa* PA14 supernatant up to 12 h after the addition.

The mixtures of **P2[Eu]** in the presence of supernatant from cultures of *P. aeruginosa* strains, producing LasB or the LasB-deficient mutants, were analyzed by HPLC-MS. To do so, 9 µL of the corresponding supernatants were added to a 0.5 mM **P2[Eu]** and 50 mM $CaCl_2$ solution. The solutions were kept at RT for 1 h before injection. Based on the preferential cleavage sequence of LasB, we expected **P2[Eu]** cleavage to produce **F1[Eu]** and **H-LAEDap(Naph)-NH$_2$** (**F3**) fragments.

F1[Eu]: ESI-MS (m/z): $[MH]^+$ calculated for $C_{27}H_{42}N_7EuN_7O_{13}$: 826.21; found 826.20 $[M+H]^+$, 413.61 $[M+2H]^{2+}$.

F3: ESI-MS (m/z): $[MH]^+$ calculated for $C_{29}H_{36}N_6O_8$: 597.27; found 597.32 $[M+H]^+$, 1193.49 $[2M+H]^+$.

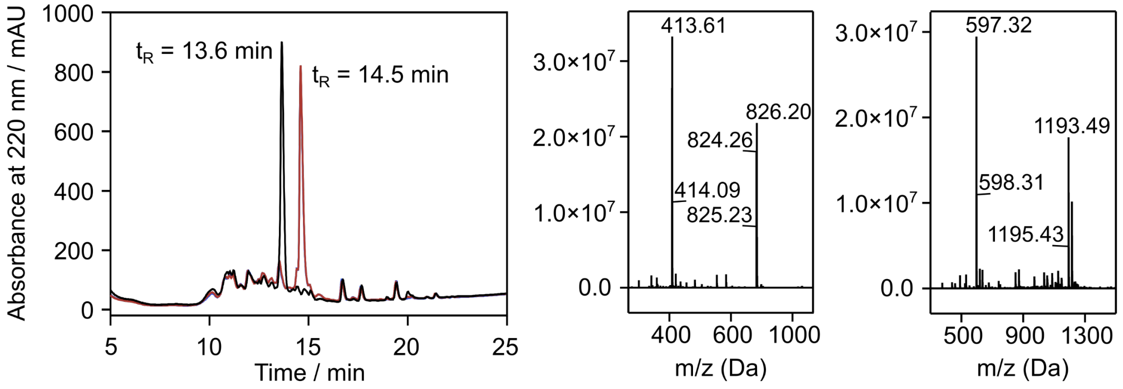

Figure S24. Overlapped HPLC chromatograms at 220 nm of a **P2[Eu]** and $CaCl_2$ solution in the presence of the *P. aeruginosa* supernatants from the wild-type (—), LasB1 mutant (—), and LasB2 mutant (—) strains (left), being the peak at t_R = 14.5 min identified as **P2[Eu]**. ESI-MS spectra of **F1[Eu]** (centre), identified at t_R = 10.6 min in the TIC, and the t_R = 13.6 min peak identified as **F3** (right).

Luminescence spectra of P2[Eu] in the presence of HLE, trypsin, and an *E. coli* supernatant

To 1 mL of a 5 µM **P2[Eu]** solution in HEPES buffer, 0.5 µL of a 67.8 µM HLE solution in 50 mM NaOAc, 200 mM NaCl, pH 5.5 (final concentration 1 µg/mL, 33.9 nM, 0.0068 equiv.) were added. The final mixture was stirred at 37 ºC and 70 rpm for 20 h at RT. Time-gated emission spectra were recorded at 37 ºC immediately and 20 h after enzyme addition using *setup 1* parameters.

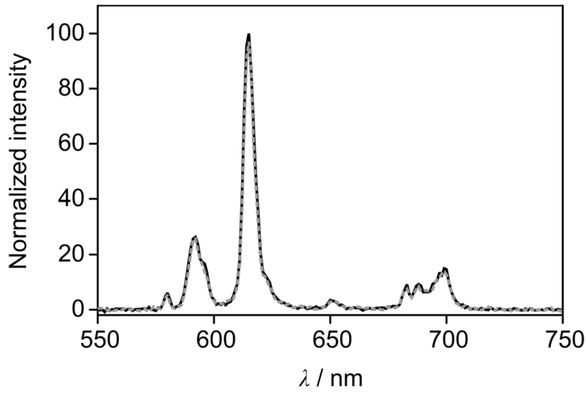

Figure S25. Time-gated emission spectra recorded at 37 ºC of a 5 µM **P2[Eu]** solution in HEPES buffer immediately (—) and 20 h after the addition (---) of HLE (1 µg/mL final concentration).

To 1 mL of a 10 µM solution of **P2[Eu]** and 1 mM $CaCl_2$ (100 equiv.) in HEPES buffer, 3.5 µL of a 41 µM solution of trypsin (3.5 µg/mL final concentration, 0.014 equiv.) were added. The final solution was stirred at 70 rpm for 20 h at RT. Time-gated emission spectra were recorded at 25 ºC immediately and 20 h after enzyme addition using *setup 1* parameters.

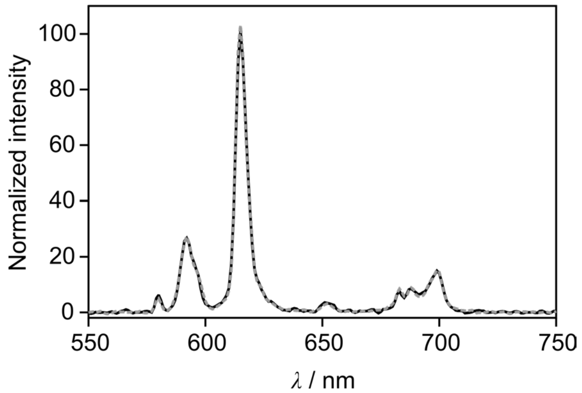

Figure S26. Time-gated emission spectra recorded at 25 ºC of a 10 µM **P2[Eu]** and 1 mM $CaCl_2$ solution in HEPES buffer immediately (—) and 20 h after the addition (---) of 3.5 µg/mL trypsin.

To 1 mL of a 10 µM solution of **P2[Eu]** and 1 mM $CaCl_2$ (100 equiv.) in HEPES buffer, 10 µL of a supernatant from an *E. coli* culture were added. The final solution was stirred at 70 rpm for 20 h at 37 ºC. Time-gated emission spectra were recorded at 37 ºC immediately and 20 h after the supernatant addition using *setup 1* parameters.

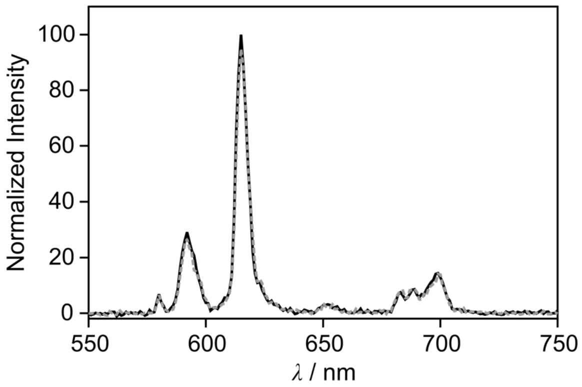

Figure S27. Time-gated emission spectra recorded at 37 ºC of a 10 μM **P2[Eu]** and 1 mM $CaCl_2$ solution in HEPES buffer immediately (—) and 20 h after the addition (---) of the *E. coli* supernatant.

Luminescence spectra of P2[Eu] in the presence of GSH and H_2O_2

To 900 μL of a 5.5 μM solution of **P2[Eu]** in HEPES buffer, 100 μL of a 1 mM solution of GSH in water (final concentration 100 μM, 20 equiv.) were added. Time-gated emission spectra were recorded at 37 ºC before and 20 h after the addition using *setup 1* parameters.

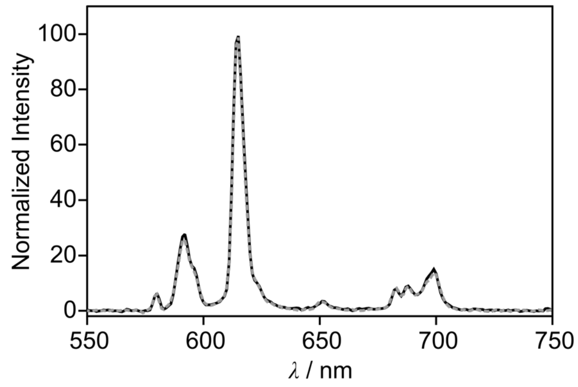

Figure S28. Time-gated emission spectra recorded at 37 ºC of a 5 μM **P2[Eu]** solution in HEPES buffer before (—) and 20 h after the addition (---) of GSH.

To 1 mL of a 5 μM solution of **P2[Eu]** in HEPES buffer, 1 μL of a 500 mM solution of H_2O_2 in water (final concentration 0.5 mM, 100 equiv.) were added. Time-gated emission spectra were recorded at 37 ºC before and 20 h after the addition using *setup 1* parameters.

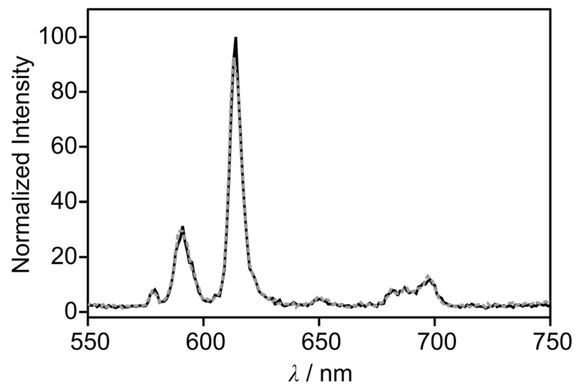

Figure S29. Time-gated emission spectra recorded at 37 ºC of a 5 μM **P2[Eu]** solution in HEPES buffer before (—) and 20 h after the addition (---) of H_2O_2.

Luminescence spectra of P2[Eu] in the presence of pyocyanin and *P. aeruginosa* supernatant

To 1 mL of a 5 µM solution of **P2[Eu]** in HEPES buffer, 1 µL of a 1 mM solution of pyocyanin in ethanol (final concentration 1 µM, 0.2 equiv.) were added. Time-gated emission spectra were recorded at 37 ºC before, immediately and 20 h after the addition using *setup 1* parameters.

To a solution containing 5 µM solution of **P2[Eu]** and 1 µM pyocyanin in HEPES buffer, 0.5 µL of a 1 M $CaCl_2$ solution and 6.5 µL of the supernatant from the *P. aeruginosa* strain culture producing LasB were added. The time-gated emission spectrum was recorded at 37 ºC 20 h after the addition using *setup 1* parameters.

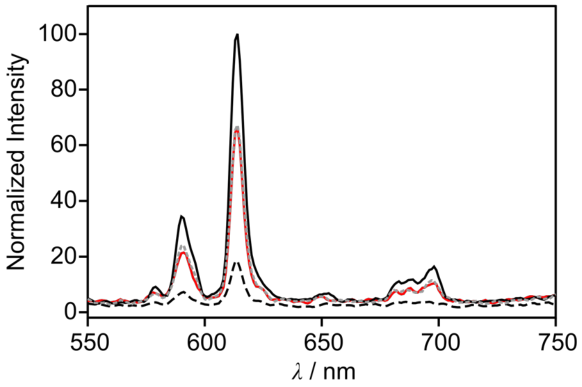

Figure S30. Time gated emission spectra recorded at 37 ºC of a 5 µM **P2[Eu]** solution in HEPES buffer before (—), immediately (—), and 20 h after (---) the addition of pyocyanin. The time-gated emission spectrum of a solution containing 5 µM **P2[Eu]**, 1 µM of pyocyanin, 0.5 mM $CaCl_2$, and 6.5 µL of the supernatant from the *P. aeruginosa* strain producing LasB at 37 ºC is shown as a dashed black line (---).

Luminescence spectra of P2[Eu] in the presence of 10% FBS and *P. aeruginosa* supernatant

To 900 µL of a **P2[Eu]** solution in HEPES buffer, 100 µL of FBS were added (final concentration 10% v/v FBS and 10 µM **P2[Eu]**). Time-gated emission spectra were recorded every hour for 12 h at 37 ºC with magnetic stirring at 70 rpm using *setup 1* parameters. Subsequently, 1 µL of a 1 M $CaCl_2$ solution (100 equiv.) and 12.5 µL of supernatant from the *P. aeruginosa* strain producing LasB were added to the solution. Time-gated emission spectra were recorded again every hour for 12 h at 37 ºC with magnetic stirring at 70 rpm using *setup 1* parameters.

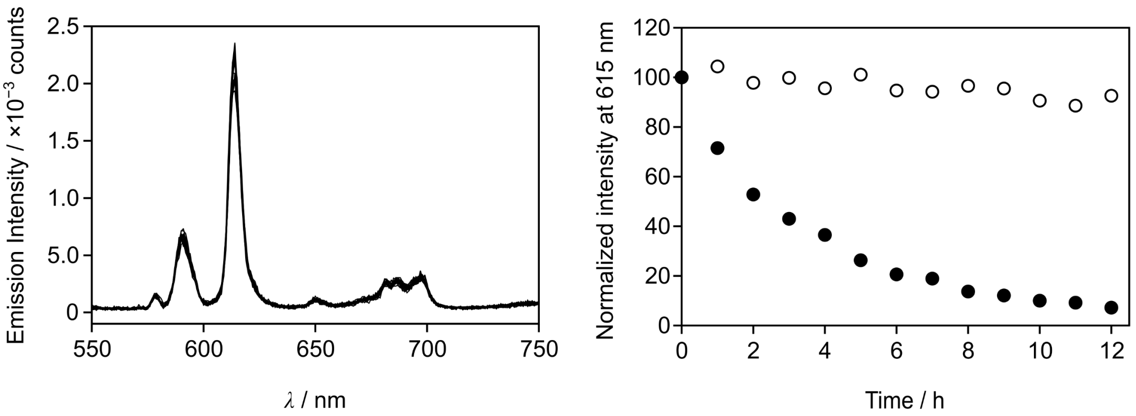

Figure S31. Luminescence spectra recorded at 37 ºC of 10 μM **P2[Eu]** in HEPES buffer in the presence of 10% v/v FBS up to 12 h after the addition (left). Time courses of 10 μM **P2[Eu]** and 10% v/v FBS (○), and 10 μM **P2[Eu]**, 1 mM $CaCl_2$, 10% v/v FBS and the wild-type *P. aeruginosa* strain supernatant (●) at 37 ºC (right).

Steady-state vs time-gated luminescence spectra of P2[Eu] in the presence of the supernatant from a LasB-deficient *P. aeruginosa* strain culture

To 790 μL of a 10 μM solution of **P2[Eu]** and 1 mM $CaCl_2$ (100 equiv.) in HEPES buffer, 10 μL of the *P. aeruginosa* LasB2 mutant supernatant were added. Time-gated and steady-state emission measurements were recorded at 37 ºC using *setup 1* and *2* parameters, respectively.

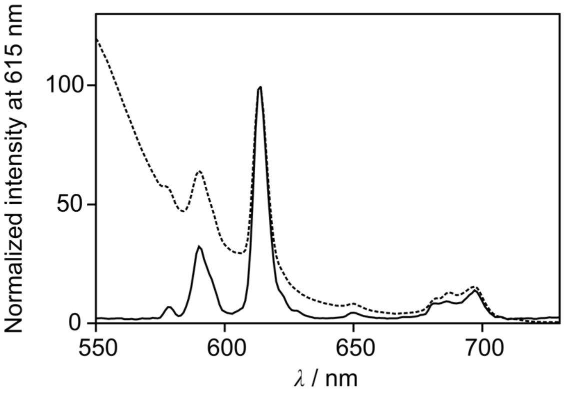

Figure S32. Time-gated (—) and steady-state (---) emission spectra at 37 ºC of a **P2[Eu]** and 1 mM $CaCl_2$ solution in HEPES buffer in the presence of the supernatant from the LasB2 mutant strain of *P. aeruginosa.*

References

(1) Proteins. In *Handbook of Biochemistry and Molecular Biology*; Fasman, G. D., Ed.; CRC Press: Cleveland, Ohio, 1976; pp 183–203.

(2) Bonnet, C. S.; Devocelle, M.; Gunnlaugsson, T. Luminescent Lanthanide-Binding Peptides: Sensitising the Excited States of Eu(III) and Tb(III) with a 1,8-Naphthalimide-Based Antenna. *Org. Biomol. Chem.* **2012**, *10* (1), 126–133.

(3) Grace, A.; Sahu, R.; Owen, D. R.; Dennis, V. A. Pseudomonas Aeruginosa Reference Strains PAO1 and PA14: A Genomic, Phenotypic, and Therapeutic Review. *Front. Microbiol.* **2022**, *13*, 1023523.

(4) Liberati, N. T.; Urbach, J. M.; Miyata, S.; Lee, D. G.; Drenkard, E.; Wu, G.; Villanueva, J.; Wei, T.; Ausubel, F. M. An Ordered, Nonredundant Library of Pseudomonas Aeruginosa Strain PA14 Transposon Insertion Mutants. *Proc. Natl. Acad. Sci.* **2006**, *103* (8), 2833–2838.

(5) De La Reberdière, A.; Lachaud, F.; Chuburu, F.; Cadiou, C.; Lemercier, G. Synthesis of a New Family of Protected 1,4,7,10-Tetraazacyclododecane-1,4, 7-Triacetic Acid Derivatives with Thioctic Acid Pending Arms. *Tetrahedron Lett.* **2012**, *53* (45), 6115–6118.

(6) Sánchez‐Fernández, R.; Sánchez‐Temprano, A.; Esteban‐Gómez, D.; Pazos, E. Probing Tyrosine Nitration with a Small Tb III ‐Metallopeptide. *ChemBioChem* **2023**, *24* (13), e202300072.

(7) Bonnet, C. S.; Devocelle, M.; Gunnlaugsson, T. Structural Studies in Aqueous Solution of New Binuclear Lanthanide Luminescent Peptide Conjugates. *Chem. Commun.* **2008**, 4552–4554.

(8) Schwartz, P. A.; Kuzmic, P.; Solowiej, J.; Bergqvist, S.; Bolanos, B.; Almaden, C.; Nagata, A.; Ryan, K.; Feng, J.; Dalvie, D.; Kath, J. C.; Xu, M.; Wani, R.; Murray, B. W. Covalent EGFR Inhibitor Analysis Reveals Importance of Reversible Interactions to Potency and Mechanisms of Drug Resistance. *Proc. Natl. Acad. Sci.* **2014**, *111* (1), 173–178.

(9) Kuzmič, P. Program DYNAFIT for the Analysis of Enzyme Kinetic Data: Application to HIV Proteinase. *Anal. Biochem.* **1996**, *237* (2), 260–273.

(10) Holstein, C. A.; Griffin, M.; Hong, J.; Sampson, P. D. Statistical Method for Determining and Comparing Limits of Detection of Bioassays. *Anal. Chem.* **2015**, *87* (19), 9795–9801.

(11) Huyke, D. A.; Ramachandran, A.; Bashkirov, V. I.; Kotseroglou, E. K.; Kotseroglou, T.; Santiago, J. G. Enzyme Kinetics and Detector Sensitivity Determine Limits of Detection of Amplification-Free CRISPR-Cas12 and CRISPR-Cas13 Diagnostics. *Anal. Chem.* **2022**, *94* (27), 9826–9834.
